# Effect of in vitro exposure of first-line antiretrovirals on healthy human spermatozoa on kinematics and motility

**DOI:** 10.1007/s11255-024-04340-x

**Published:** 2025-01-03

**Authors:** Sohan Zane Pinto, Natalie Aneck-Hahn

**Affiliations:** 1 Department of Urology, Faculty of Health Sciences, Steve Biko Academic Hospital, University of Pretoria, Pretoria, South Africa; 2 Environmental Chemical Pollution and Health Research Unit, University of Pretoria Faculty of Health Sciences, Pretoria, South Africa

**Keywords:** Antiretrovirals, Dolutegravir, Tenofovir, Emtricitabine, Sperm kinematics, Sperm motility, Male infertility

## Abstract

**Purpose:**

Contemporary antiretroviral (ARV) medications are used by millions of men for HIV treatment worldwide. Limited data exist on their direct effect on sperm motility. This pilot study hypothesizes that in vitro exposure to ARVs will reduce sperm kinematic and motility parameter values.

**Methods:**

This laboratory-based experimental study analyzed sperm motility and kinematics after exposure to the ARVs Dolutegravir, Tenofovir, and Emtricitabine, individually and in combination. Each participant (n = 23) served as their experimental control. The Microptic SCA® Computer Assisted Sperm Analysis (CASA) system, Barcelona, Spain was used to generate quantitative data on sperm motility and the kinematics Straight-line velocity (VSL), Straightness index (STR), Linearity Index (LIN), Beat cross frequency (BCF), and the oscillation index (WOB).

**Results:**

VSL, STR, LIN, and WOB of the non-progressive (grade c) spermatozoa were significantly decreased after ARV treatment. BCF of the medium velocity progressive sperm population (grade b) was significantly increased 90 min after exposure in the Tenofovir arm, and a significant decrease in the proportion of grade b spermatozoa was recorded at 90 min in all the antiretroviral arms when compared to the control arm. No impaired sperm motility was observed within the first 30 min of exposure.

**Conclusion:**

Pharmacovigilance is a healthcare emergency as the fast-changing world of newer drugs leaves clinicians vulnerable. They must prescribe drugs whose long-term somatic and germline adverse effects are not fully understood. Guidelines and drugs are changing faster than we can monitor for side effects. Despite Dolutegravir being the only mainstream integrase inhibitor first-line ARV in South Africa for five years, its replacement, Cabotegravir, is already being launched. More research in this field is required, especially for commonly prescribed drugs. This preliminary pilot study concludes that the current first-line ARVs used by HIV patients and HIV-negative patients on pre-exposure prophylaxis (PrEP) can alter sperm motility and kinematics. Further research with a larger sample size is warranted to quantify its impact on human fertility, addressing the limitations of this study, before a comprehensive conclusion of the effects of ARVs on human male fertility can be drawn. Of particular importance would be to study the impact of ARVs on reactive oxygen species levels in semen and sperm DNA fragmentation.

## Background

### Introduction

A major public health issue globally is the Human Immunodeficiency Virus (HIV) infection pandemic, which is more prevalent in middle- and low-income countries. South Africa has the largest number of people living with HIV [1]. Several advances in HIV treatment in recent years include the roll-out of combination three-in-one first-line ARV pills and the pre-exposure prophylaxis (PrEP) pill. The World Health Organization and National Department of Health of the Republic of South Africa are determined to fast-track the roll-out of newer ARVs to halt new infections and control the pandemic that has been raging for 4 decades [2–6].

Currently, patients on Efavirenz (EFV)-containing fixed-dose combination pills are being transitioned to Dolutegravir (DTG)-containing fixed-dose combination pills, owing to better viral suppression and improved patient compliance with DTG. Women of childbearing age are counseled about the potential risks of neural tube birth defects when taking DTG. The new fixed-dose combination pill includes DTG, Lamivudine (3TC), and Tenofovir (TFV). Tenofovir is also used in PrEP together with Emtricitabine (FTC) [2–4].

There have been concerns about the effect of ARVs on human semen parameters [7]. A recent study showed a significant increase in sperm deoxyribonucleic acid (DNA) fragmentation after initiation of combination antiretroviral therapy [8]. Most studies focusing on the effect of ARVs on human sperm motility have been limited to older ARVs. Some older ARVs, such as Efavirenz and Saquinavir, are associated with impaired sperm motility [9, 10]. No semen motility studies have been performed with FTC, while TFV has only been studied in vivo by analyzing semen from patients on TFV-based combination therapy. Confounding factors, such as age, smoking exposure, comorbid status, nutritional status, chronic medication use, dietary patterns, body mass index, and co-infections with other sexually transmitted diseases, are known to affect various semen parameters, often in conflicting ways that make interpretation of in vivo study results difficult. To correct for this, in vitro studies may be undertaken where the effects of ARVs on the motility of previously unexposed, healthy spermatozoa can be studied in isolation. The focus on decreasing HIV transmission via infected semen has provided data documenting the therapeutic levels of contemporary ARVs in the semen of users, using validated liquid chromatography tandem mass spectrometry methods [11–15], but very limited research has looked into what these therapeutic drug levels due to the male human gamete.

### Evolution of antiretroviral therapy

In the last three decades, more than 25 ARVs have been developed for HIV targeting antiretroviral therapy. Treatment guidelines have changed frequently and monotherapy has given way to combination therapy, including once-daily oral fixed-dose combination pills. The ever-changing landscape of antiretroviral therapy is guided not only by the financial costs, drug efficacy, and side effects of ARVs; but also to the viral resistance patterns and patient adherence to ARV regimens [16]. Growing viral resistance to first-line ARVs, such as Efavirenz (EFV) and Nevirapine, are forcing governments to move swiftly to newer agents [17, 18].

One such newer ARV that can replace EFV is DTG, which is combined with 3TC and TFV as a single daily pill, abbreviated as TLD. Dolutegravir is an inexpensive highly potent ARV with good tolerability, but, most importantly, it has a higher genetic barrier to developing HIV drug resistance [4, 6]. Delays in the roll-out of DTG was because of fears of possible teratogenicity and neural tube birth defects during pregnancy [19, 20]. The benefits of DTG to women of childbearing age greatly outweigh the risks, so treatment guidelines now recommend DTG as a first-line therapy to all women, provided that they have been counseled about the low potential risks for birth defects in pregnancy [4, 5, 21].

### Antiretroviral therapy and male fertility

As the antiretroviral landscape evolves rapidly, little is known about the effects of the newer ARVs on male fertility. From multiple studies on men taking ARVs that have recently fallen into disuse, we know that semen quality is affected by decreased semen volume, sperm count and motility, and increased abnormal morphology documented [22–26]. Increased sperm DNA fragmentation and a decrease in sperm mitochondrial DNA copies have also been documented in men taking ARVs [27, 28].

In an in vitro study of the effects of ARVs on semen, saquinavir (SQV) was shown to decrease sperm mitochondrial potential in a time- and dose-dependent manner [10]. In a decade-long prospective cohort study, a significant association was found between impaired sperm motility and EFV use in men [29]. Efavirenz may also contribute to male infertility by elevating sex hormone-binding globulin (SHBG) levels, which in turn induces hypogonadism [30]. In animal studies, Lopinavir/Ritonavir has been shown to impair semen parameters and cause oxidative damage to the testis [31].

The results from TFV and FTC are conflicting. Laboratory-based animal studies and one in vitro study with human semen have associated these two ARVs with sperm immobility [32–34]. However, human trials with men and women on PrEP (TFV + FTC) have shown non-significant changes in fertility and pregnancy outcomes of the ARV group compared with the placebo group [35, 36]. It is worth noting that in human trials, it may be difficult to draw conclusions on semen parameter values from pregnancy outcomes of study participants. Semen parameters are known to be altered by a multitude of anatomical, genetic, medical, environmental, and occupational factors [37]. Age, dietary patterns, alcohol intake, caffeine intake, body mass index (BMI), cigarette exposure, sexually transmitted infections (STI), and the presence of varicoceles in study participants are just a few of the several well-documented variables that affect semen quality [38–46]. Using an in vitro experimental study on spermatozoa from the same participant (as his own control), many of these variables can be eliminated. Changes in motility measured by computer-aided semen analysis (CASA) can be directly correlated with male fertility potential and warrants an investigation for all newer ARVs [47, 48].

### Quantifying ARV levels in human semen

It is known that the male genital tract may be a body compartment for HIV to replicate and develop resistance in, and that the ability of ARVs to penetrate into, and be concentrated in, semen can be beneficial by decreasing both HIV transmission and the development of resistance [49, 50]. This has led to several studies that quantified the ARV levels in human semen when patients took the regular recommended oral dosages of the same.

ARVs exist in their free, unmetabolized form in seminal plasma. Cells take up free ARVs from plasma and phosphorylate the ARVs intracellularly into their active form. Therefore, free TFV and free 3TC in seminal plasma exist as TFV-diphosphate and 3TC-triphosphate inside cells. Emtricitabine (FTC) is structurally similar to 3TC and is phosphorylated to FTC-triphosphate, which has a longer elimination half-life and is preferred when compliance is a problem [51, 52]. Both FTC and 3TC may be used interchangeably clinically [51, 53]. Some ARVs, which are highly bound to blood plasma proteins, are bound significantly less to seminal plasma proteins. For DTG, which in blood plasma exists as only a 0.45% free (unbound) form, in seminal plasma exists in a 48% free (unbound) fraction [13, 14, 54–56].

Tenofovir has been documented to penetrate into seminal plasma rapidly and achieve extracellular concentrations of 162–483 ng/mL. In seminal plasma, it can reach 19 times the concentration in blood plasma [11, 13]. Lamivudine can reach extracellular concentrations in seminal plasma more than 6 times those in blood plasma, with a documented range of 1460–4320 ng/mL [14, 57]. Structurally similar FTC also reaches higher concentrations in seminal plasma, of between 258 and 3687 ng/mL [12, 13, 58, 59]. Dolutegravir can reach total concentrations of 39–423 ng/mL and unbound concentrations of 13–203 ng/mL in seminal plasma [15, 56].

### Aim and objectives

This is a pilot study of the fertility implications of contemporary ARVs used by millions of men worldwide. Sperm motility and kinematics were analyzed during exposure to ARVs, both individually and in combination, and compared with the negative control (unexposed spermatozoa).

## Methods

### Ethics approval and consent to participate

Approval was granted by the Research Ethics Committee of the Faculty of Health Sciences, University of Pretoria-Reference number 16/2021. The participants who volunteered remained anonymous and were allocated a study number. All contact sessions with them were in private. There was no cost to the volunteer. All the information from the volunteer was kept confidential. If medical abnormalities at screening were detected, the volunteer was informed and referred for treatment if appropriate. The blood for HIV serology was collected before semen donation, to limit inconvenience to the donor. Results were made available to the volunteers upon request. Simple language was used to explain the study and particulars of participation.

### Study setting and design

The study was conducted at the Andrology Clinic at the Department of Urology of Steve Biko Academic Hospital. This was a laboratory-based experimental study. Informed consent was obtained before enrollment of participants in the study.

Sperm motility can be affected by environmental, lifestyle, and medical factors listed in the exclusion criteria of Table 1, as well as showing inter-participant variability. Healthy, presumably unexposed motile spermatozoa for the experiments were selected through a three-step procedure to minimize the effect of confounding factors. Step one was a screening questionnaire to the volunteers, and HIV testing discussed in the section “Participant screening questionnaire”. The participants who met the eligibility criteria went on to Step two of a basic semen analysis described in the section “Semen collection and basic semen analysis”. Step three involved a double-layer centrifugation process to remove immotile spermatozoa, debris, and potential impurities from the semen sample described in the section “Sperm preparation”. The experiments were performed on the prepared spermatozoa in vitro in a standardized manner by the same team, and the Microptic SCA® CASA system (Barcelona, Spain) was used to generate standardized quantitative data on sperm motility and kinematics with high precision in a reproducible manner. A cross-over randomized-controlled trial design ensured that the exposed spermatozoa were compared with the unexposed control spermatozoa from the same study participant.

**Table 1:** Participant selection: inclusion and exclusion criteria

Inclusion criteria		Exclusion criteria	
Sex	Male	Social habits	Tobacco user, smokers, marijuana users, recreational drug users, and moderate or heavy alcohol consumption (more than 40 g per day) [43, 44]
Age	18–30 years [41]	Chronic medication	Antihypertensives, diabetic medication, antiretrovirals, anticoagulants, antiepileptics, antipsychotics
Sexual history	Minimum 2-day and a maximum 3-day history of abstinence, not on PrEP (pre-exposure prophylaxis)	Chronic conditions	Cardiovascular disease, heart attacks, stroke, diabetes, positive HIV (Human Immunodeficiency Virus) status, tuberculosis, epilepsy, psychosis
Semen volume	At least 1.5 ml	Other conditions	Previous or current sexually transmitted infection, Body Mass Index more than 25 [39, 42, 45, 46]
Semen parameters	Normal range of semen parameter values as per the World Health Organization (WHO) 2010	Other Medication	Use of herbal supplements, fat burners, anabolic steroids, corticosteroids, vitamin supplements, or anti-inflammatories within 4 weeks of sample collection [37, 38]

### Participant screening questionnaire

A questionnaire was used to screen for lifestyle factors and medical conditions that affect human semen parameters. The participants were screened for chronic medical conditions (self-reported) and exposure to specific medications (self-reported), and were offered voluntary HIV counseling and testing. The participant selection took place according to the criteria summarized in Table 1.

### Semen collection and basic semen analysis

All semen samples were collected into a standard sperm-collection cup at the Andrology department of Steve Biko Academic Hospital in a private room. The sample was collected from the volunteers provided that they met the inclusion criteria of having a minimum of 2 days and a maximum of 3 days of sexual abstinence. All volunteers were given clear verbal and written instructions on how to collect the specimen with emphasis on collecting a complete sample. All samples were collected by masturbation and ejaculation into a clean wide-mouthed, non-spermicidal plastic container that was labeled and incubated at 37^0^C for 30 to 40 min to allow the semen to liquefy. Once liquefied, a standard semen analysis was done according to WHO 2010 guidelines, which starts with the assessment of seminal physical characteristics, including appearance, viscosity, volume, and pH. Sperm numbers and concentration were checked in Marienfeld Neubauer's improved bright-line haemocytometer at 40 × magnification with trypan blue stain to highlight the sperm heads. Sperm motility was assessed on a wet preparation by placing 22 μl semen in a prewarmed 20-μm-deep, two chamber Leja® counting chamber (Leja Products B.V., Nieuw-Vennep, The Netherlands) and using the Microptic Sperm Class Analyzer (SCA) CASA system (Barcelona, Spain). The specimen was screened for leucocytes, erythrocytes, bacteria, and cell agglutinates. Round cell counts had to be under 5 million/mL and leucocytes under 1 million/mL for enrollment in the study. As total motility levels below 50% excluding the sample from the study, vitality studies, mixed anti-globulin reaction test, and sperm morphology smears were not a part of screening [60–63].

### Sperm preparation

While an aliquot of the liquefied and mixed semen was screened, the remainder was prepared to increase the yield of functionally and morphologically normal spermatozoa and to separate debris, lymphocytes, dead spermatozoa, reactive oxygen species, and non-germ cells from the live spermatozoa. This process improves sperm survival time and for this study provided standardized semen parameters for the control and test samples. Spermatozoa were prepared within 1 h of ejaculation by the author and andrology laboratory support staff. It ran parallel to the screening process to prevent any delay [61, 64].

From 2.5 to 3 mL liquefied and mixed semen was placed over equal proportions of Vitromed V Grad 40® and Vitromed V Grad 80® (Vitromed GmbH, Jena, Germany) in a 15 mL Falcon centrifuge tube, which was centrifuged at 300 × g for 20 min in a bench centrifuge (Eppendorf Centrifuge 5804R, Eppendorf Ibérica SLU, Madrid, Spain). The supernatant was discarded and the sperm pellet washed with 5 ml Kitazato® Gamete Buffer (Kitazato Corporation, Shizuoka, Japan) and centrifuged at 200 × g for 5 min to remove particles of colloidal silica. The final sperm pellet obtained was re-suspended in a new tube by gentle pipetting with Vitromed V Sperm Wash® (Vitromed GmbH, Langenfeld, Germany), containing Human Serum Albumin (HSA) 5 mg/mL; then, sperm concentration and motility were determined. Eleven microliters of this sperm suspension was placed in a Leja® counting chamber to assess concentration and motility. Calculations were made and further dilutions in buffer made to achieve four test aliquots of 200 µL with a concentration of 10–20 million sperm/200 µL buffer [61, 65, 66].

### Preparation of working drug solutions

The study arm entailed four prepared sperm aliquots of 200 µL that were diluted in 800 µL working solutions of the ARVs under study. The final samples for the sperm motility experiments comprised 1 mL mixture of 10–20 million spermatozoa in sperm-friendly buffer, and the ARV in the desired concentration. One sample served as the negative control and was diluted in 800 µL buffer only, and the remaining three were diluted in 800 µL DTG, TFV, or DTG + FTC + TFV working solutions. Rounding the concentrations to the nearest 100 ng/mL ensured standardized solutions that could be prepared frequently during the study. For DTG and TFV, the final concentration was 300 ng/mL; FTC, 3000 ng/mL. All the standardized concentrations are well within the documented limits in the literature.

Stock solutions: Fresh batches of pure Active Pharmacological Ingredients (API) working solutions were prepared each day of the study. Certified reference standards of the APIs were purchased from Clearsynth, Mumbai, India. Stock solutions of the API were kept in the laboratory refrigerator. As the drugs were stored in the laboratory as high concentration stock solutions, they were diluted to working drug solutions to achieve the desired final concentrations. Dolutegravir and Emtricitabine are soluble in 95% methanol, while Tenofovir is soluble in water. Dolutegravir and Tenofovir were stored as 1 mg/mL concentration stock solutions, while Emtricitabine was stored as a 15 mg/mL stock solution. Because of daily dilution of the API stock, the final methanol concentration the semen specimens were exposed to was 0.03%. Methanol is not injurious to spermatozoa in concentrations as high as 15% and is commonly used in semen cryopreservation [10, 67–72].

Working solutions: To prepare the working drug solution of DTG, 10 µL of the 1 mg/mL DTG stock solution was mixed with 10 mL Kitazato® Gamete Buffer (final concentration1 µg/mL); 300 µL of this was mixed with 500 µL buffer; and later with 200 µL of prepared sperm suspension in buffer (final concentration 300 ng/mL). The same calculations were used to prepare the 300 ng/mL TFV working solution from its 1 mg/mL its aqueous stock solution. For the ARV combination working solution, a 15 mg/mL FTC in methanol stock solution was diluted 1:1000 by dissolving 10 µL in 10 mL Kitazato® Gamete Buffer (final concentration 15 µg/mL FTC); 200 µL of this was mixed with 200 µL prepared spermatozoa, 300 µL 1 µg/mL DTG, and 300 µL 1 µg/mL TFV, and the resultant combination ARV solution had 300 ng/mL DTG + 300 ng/mL TFV + 3000 ng/mL FTC per ml of working solution. This mimics the combination ARV levels expected in the semen of a user of the new fixed-dose combination tablet. Emtricitabine was only studied in combination as it is never used as monotherapy in clinical practice.

### Exposure of prepared spermatozoa to working solutions

The four prepared sperm samples entered the study arms with each containing 200 µl containing 10–20 motile million spermatozoa. Each aliquot was then mixed with 800 µL of pre-prepared drug working solutions to achieve a final concentration of 10–20 million sperm/mL in all the study arm samples. The negative control was mixed with 800 µL buffer. Of the remaining three samples, one was exposed to DTG working solution (final concentration 300 ng/mL) with prepared spermatozoa; another was exposed to the TFV working solution (final concentration 300 ng/mL) with prepared spermatozoa; the other was exposed to 800 µL of the combined ARV working solution (final concentration 300 ng/mL DTG + 300 ng/mL TFV + 3000 ng/mL FTC). All four specimens were incubated at 37^0^C for 2 h. Samples of 33 µL were taken from each specimen into a Leja® counting chamber at 0, 30, 60, and 90 min after exposure for motility assessment [10, 67, 68].

### Motility assessments on Microptic® Sperm Class Analyzer

High-precision quantitative data on sperm motility and kinematics were documented for all 4-study arm aliquots at 30-min intervals. The Microptic Sperm Class Analyzer (SCA) CASA system version 6.5.0.91 was initiated, and the database was opened. The sample identification details were entered and saved, and a new spermiogram initiated. The microscope stage and the 20 µm-deep, four-chamber Leja® counting chambers (Leja Products, The Netherlands) were prewarmed to 37 °C. Thereafter, 11 µl from each of the study aliquots was loaded into the Leja® counting chambers. Live video mode at 50 frames per second was initiated to perform data acquisition. A minimum of 200 and up to 500 motile spermatozoa were assessed in five or more microscopic fields per chamber under 200 × magnification. Curvilinear Velocity (VCL) is a measure of cell vigor and measures the average velocity of a sperm head along its curvilinear path. Straight-line Velocity (VSL) measures the average velocity traveled by a sperm head along a straight-line path that joins the positions it was first and last detected in Average Path Velocity (VAP) is calculated from the average path obtained by smoothing the curvilinear path of the sperm head. Amplitude of Lateral Head displacement (ALH) measures the magnitude of displacement of the sperm heads from the average path on one side. Finally, Beat Cross frequency (BCF) measures the rate at which the curvilinear path crosses the average path [61]. The derived values of straightness index (STR) which is calculated as VSL/VAP, linearity index (LIN) which is VSL/VCL, and oscillation index/wobble (WOB) which is VAP/VCL are expressed as percentages [73, 74].

**Fig. 1:**
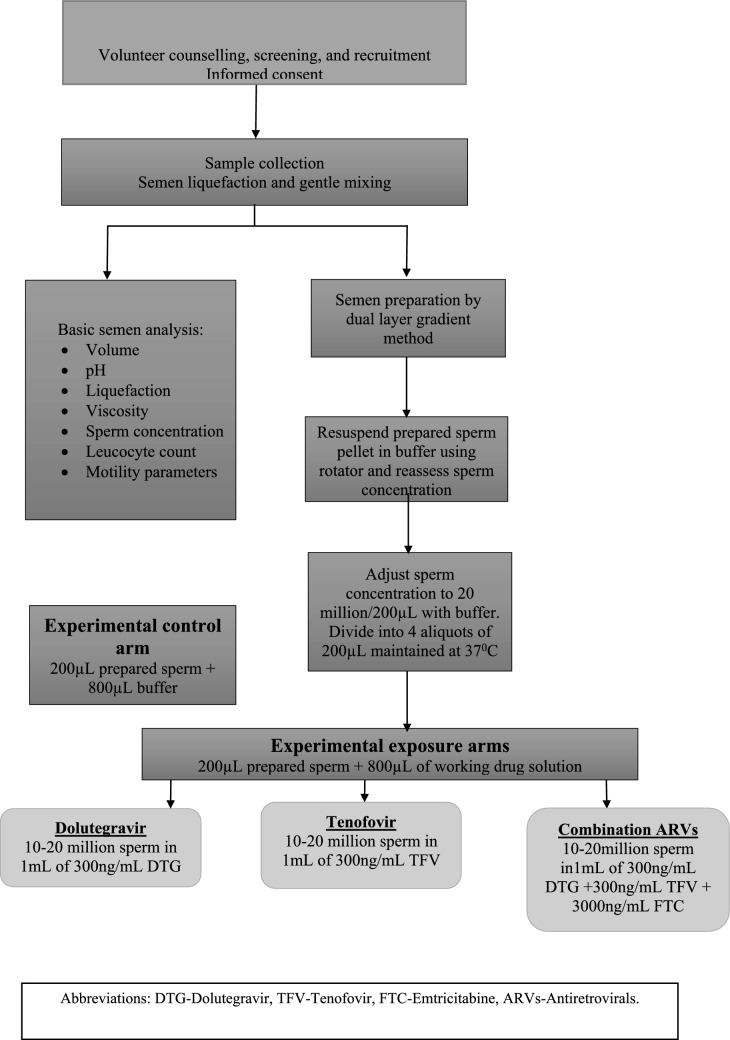
Flow diagram summarizing the workflow that was followed during this study. *DTG* Dolutegravir, *TFV* Tenofovir, *FTC* Emtricitabine, *ARVs* Antiretrovirals

**Fig. 2:**
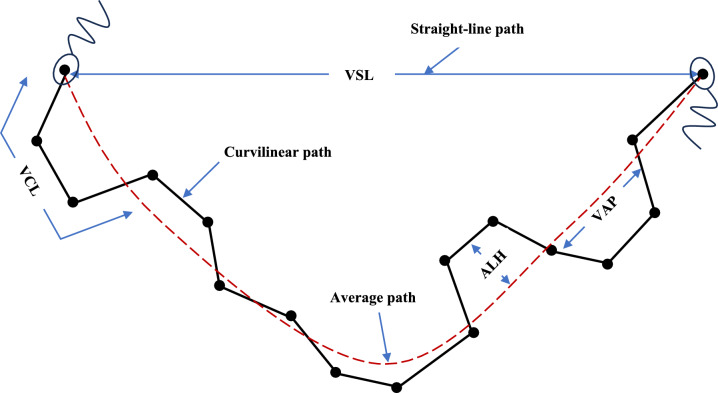
Diagram demonstrating CASA parameters. *VCL* curvilinear velocity, *VAP* average path velocity, *VSL* Straight-line velocity, *ALH* Amplitude of lateral head displacement

**Fig. 3:**
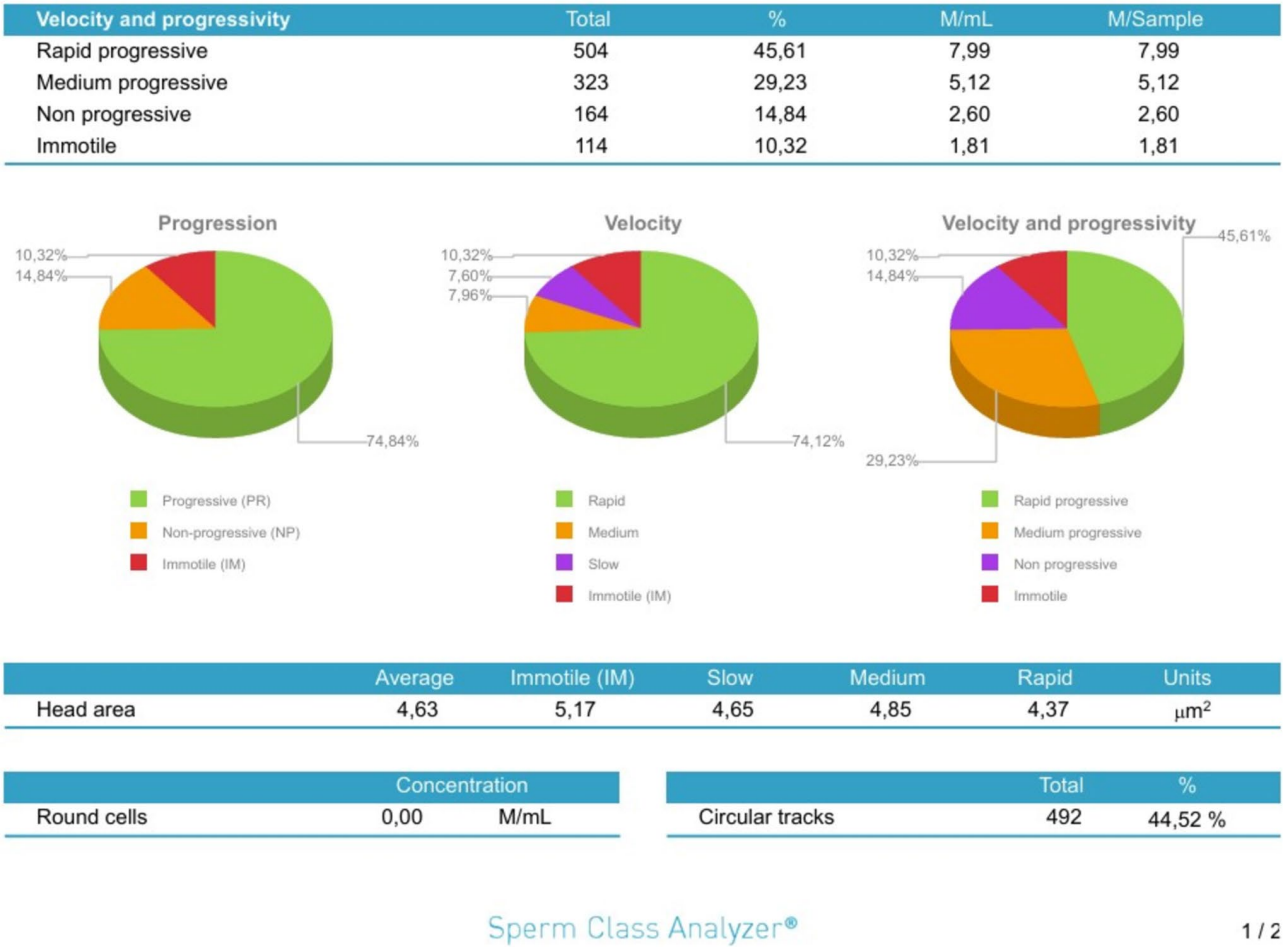

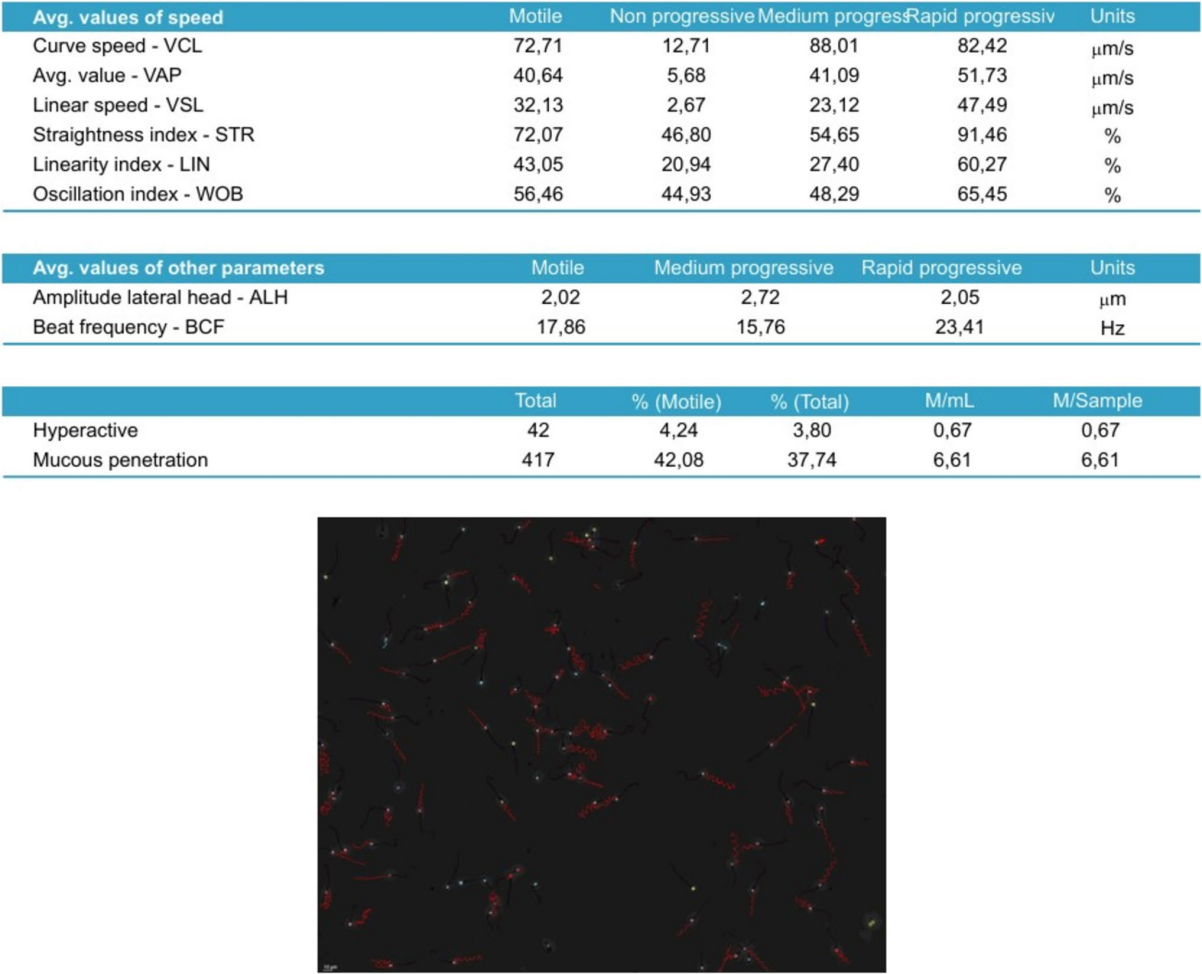
Example of spermiogram capture of observations for participant S19’s Dolutegravir exposure arm at 0 min

A total of 16 experimental arm observations were made per participant as the four aliquots per participant (Buffer only control, DTG, TFV, DTG + TFV + FTC) were assessed at four times (0, 30, 60, and 90 min). Each observation contained the percentages of the different sperm motility categories described below, and provided the average values for the multiple kinematic parameters described above. The combination arm comprising DTG + TFV + FTC is referred to as the MIX arm. Data were captured on both an Excel® sheets and Sperm Class Analyzer® spermiograms. An example of a spermiogram is shown in Figs. 1, 2, 3. The spermiogram provides average values for the above kinematic parameters for three of the four sperm motility categories: rapid progressive motile with VSL ≥ 25 μm s^−1^ (or grade a), medium progressive motile with 5 ≤ VSL < 25 μm s^−1^ (or grade b), and non-progressive motile sperm with VSL < 5 μm s^−1^ (or grade c). The picture at the bottom of the spermiogram shows a video screenshot of a grade a spermatozoa with red tracks, grade b with blue tracks, and grade c as yellow circles. The fourth category of immotile (or grade d) sperm is only measured as a percentage of the total sperm population but has no kinematic parameter. The WHO categorization of sperm motility grades reverted to the older grade a–d system after temporarily halting that terminology between 2010 and 2021 [75, 76].

### Statistical analysis of data

To assess factors associated with progression and time, we utilized linear regression, since the outcomes were continuous. A power analysis, to assess the statistical power needed to detect an effect, was done in Stata after the study was completed. Our sample size of 368 observations has 78% power, assuming a 5% significance level.

The study enrolled 23 out of the 43 volunteers who met the inclusion criteria, passed the basic semen analysis screening, and whose specimens completed the sperm preparation and exposure experiments with no errors or missing values. Mean semen parameter values at the screening of these 23 participants are shown in Table 2. Descriptive statistics were summarized using means and interquartile ranges. Sixteen observations (four treatment levels at four-time points) for each participant resulted in 368 observations in 23 participants. The data from the spermiograms were uploaded into an Excel spreadsheet and imported on STATA 17 software (Texas, USA) for subsequent analysis.

**Table 2:** Basic semen parameter values of donor samples used in this investigation (*n* = 23)

Variable	Shapiro–Wilk W test P value	Mean (SD) OR Median (IQR)
Volume (ml)	< 0.001	3.5 (2.8—5)
pH	0.001	7.5 (7.2—7.5)
Sperm count (mil/ml)	0.001	80.32 (48.5—110.79)
Sperm per sample (mil)	0.146	367.4 (262.4)
Motility a (%)	0.983	55.7 (16.3)
Motility b (%)	0.586	23.3 (8.4)
Motility c (%)	0.006	8 (3—15)
Motility (a + b + c) (%)	0.005	93 (81—97)
Progressive motility (a + b) (%)	0.225	79.1 (12.7)
Round cells (10^6/ml)	< 0.001	3.75 (1.75—10)
ENTZ pus cells(10^6/ml)	0.007	0.25 (0—0.75)

Means and standard deviations (SD) are reported for normally distributed data as per the Shapiro–Wilk test P value, while medians and interquartile ranges (IQR) are reported for non-normally distributed data

Continuous variables were used. The data from this within-participant study were analyzed by linear regression to assess factors associated with progression at times 0, 30, 60, and 90 min. Of importance was comparing treatment levels and changes from baseline to follow-up times. All tests were conducted at the 5% level of significance using Stata Release 17.

## Results

The within-participant experimental design assessed sperm motility under four treatment levels (control with buffer only, DTG, TFV, and DTG + TFV + FTC) at four times (baseline-0 min, 30 min, 60 min, and 90 min). Semen from 23 volunteers successfully passed all stages of the study including questionnaire screening, basic semen analysis, the sperm preparation procedure, and the exposure to the ARVs out of the 30 recruited. The data are presented in Tables 2 and 3.

**Table 3:** Results from linear regression models on the impact of the intervention on outcomes at the observed time intervals. Significant changes are highlighted in red

Variable description	0 min		30 min		60 min		90 min	
	Coefficient (95% CI)	P value	Coefficient (95% CI)	P value	Coefficient (95% CI)	P value	Coefficient (95% CI)	P value
Progression (%)								
Progressive (PR)								
DTG	−3.44 (−12.52; 5.64)	0.454	−0.21 (−9.4; 8.99)	0.965	−2.46 (−11.83; 6.91)	0.604	−2.61 (−11.96; 6.74)	0.580
MIX	−2.96 (−12.04; 6.12)	0.519	0.11 (−9.08; 9.31)	0.981	−3.72 (−13.09; 5.65)	0.432	−0.49 (−9.84; 8.86)	0.918
TFV	−4.49 (−13.57; 4.59)	0.328	−0.61 (−9.81; 8.58)	0.895	−4.19 (−13.56; 5.18)	0.377	−0.87 (−10.22; 8.48)	0.853
Non-progressive (NP)								
DTG	−0.23 (−4.23; 3.76)	0.907	0.15 (−3.78; 4.09)	0.938	−0.02 (−4.09; 4.05)	0.993	−0.86 (−4.9; 3.19)	0.675
MIX	−0.74 (−4.74; 3.25)	0.713	−1.03 (−4.96; 2.91)	0.606	−0.05 (−4.12; 4.02)	0.981	−1 (−5.05; 3.04)	0.623
TFV	0.93 (−3.06; 4.93)	0.644	−0.48 (−4.41; 3.45)	0.809	0.29 (−3.78; 4.36)	0.887	−1.2 (−5.25; 2.84)	0.556
Immotile (IM)								
DTG	3.67 (−2.25; 9.6)	0.221	0.05 (−5.77; 5.88)	0.986	2.48 (−3.46; 8.41)	0.409	3.47 (−2.69; 9.62)	0.266
MIX	3.7 (−2.22; 9.62)	0.218	0.92 (−4.91; 6.74)	0.755	3.77 (−2.16; 9.71)	0.210	1.49 (−4.67; 7.65)	0.631
TFV	3.56 (−2.37; 9.48)	0.236	1.09 (−4.73; 6.92)	0.710	3.89 (−2.04; 9.83)	0.196	2.08 (−4.08; 8.23)	0.505
Motile								
DTG	−3.67 (−9.6; 2.25)	0.221	−0.05 (−5.88; 5.77)	0.986	−2.48 (−8.41; 3.46)	0.409	−3.47 (−9.62; 2.69)	0.266
MIX	−3.7 (−9.62; 2.22)	0.218	−0.92 (−6.74; 4.91)	0.755	−3.77 (−9.71; 2.16)	0.210	−1.49 (−7.65; 4.67)	0.631
TFV	−3.56 (−9.48; 2.37)	0.236	−1.09 (−6.92; 4.73)	0.710	−3.89 (−9.83; 2.04)	0.196	−2.08 (−8.23; 4.08)	0.505
Velocity (%)								
Rapid								
DTG	−2.55 (−11.86; 6.75)	0.587	−0.06 (−9.41; 9.29)	0.990	−1.51 (−11; 7.98)	0.752	−0.6 (−10.21; 9.02)	0.902
MIX	−0.9 (−10.2; 8.41)	0.849	0.15 (−9.2; 9.5)	0.975	−2.83 (−12.31; 6.66)	0.555	1.68 (−7.94; 11.29)	0.730
TFV	−2.4 (−11.7; 6.91)	0.610	−0.34 (−9.69; 9.01)	0.943	−3.15 (−12.64; 6.33)	0.511	1.09 (−8.53; 10.7)	0.823
Medium								
DTG	−1.25 (−3.73; 1.23)	0.318	−0.16 (−1.7; 1.38)	0.835	−1.34 (−3.09; 0.4)	0.130	−**2.65 (**−**4.8; **−**0.5)**	**0.016**
MIX	−**2.51 (**−**4.99; **−**0.04)**	**0.047**	−0.43 (−1.97; 1.11)	0.579	−1.29 (−3.04; 0.46)	0.147	−**2.42 (**−**4.57; **−**0.28)**	**0.027**
TFV	−1.65 (−4.13; 0.82)	0.188	−0.58 (−2.11; 0.96)	0.458	−1.55 (−3.3; 0.19)	0.081	−**2.14 (**−**4.29; 0.01)**	**0.051**
Slow								
DTG	0.13 (−3.01; 3.28)	0.934	0.17 (−2.96; 3.3)	0.914	0.38 (−2.68; 3.43)	0.807	−0.22 (−3.36; 2.92)	0.890
MIX	−0.29 (−3.43; 2.86)	0.856	−0.64 (−3.77; 2.49)	0.687	0.34 (−2.71; 3.4)	0.824	−0.75 (−3.89; 2.39)	0.637
TFV	0.49 (−2.65; 3.64)	0.757	−0.18 (−3.31; 2.95)	0.910	0.81 (−2.24; 3.86)	0.599	−1.02 (−4.16; 2.11)	0.518
Immotile								
DTG	3.67 (−2.25; 9.6)	0.221	0.05 (−5.77; 5.88)	0.986	2.48 (−3.46; 8.41)	0.409	3.47 (−2.69; 9.62)	0.266
MIX	3.7 (−2.22; 9.62)	0.218	0.92 (−4.91; 6.74)	0.755	3.77 (−2.16; 9.71)	0.210	1.49 (−4.67; 7.65)	0.631
TFV	3.56 (−2.37; 9.48)	0.236	1.09 (−4.73; 6.92)	0.710	3.89 (−2.04; 9.83)	0.196	2.08 (−4.08; 8.23)	0.505
Velocity and progressivity (%)								
Rapid progressive								
DTG	−0.05 (−6.57; 6.47)	0.988	1.62 (−5.23; 8.48)	0.639	−0.23 (−7.09; 6.64)	0.948	1.06 (−6.06; 8.18)	0.769
MIX	0.71 (−5.81; 7.22)	0.830	0.5 (−6.36; 7.35)	0.886	−1.04 (−7.91; 5.82)	0.763	1.7 (−5.42; 8.81)	0.637
TFV	−0.42 (−6.94; 6.09)	0.898	1.58 (−5.28; 8.43)	0.649	−1.78 (−8.65; 5.08)	0.607	1.97 (−5.14; 9.09)	0.583
Medium progressive								
DTG	−3.39 (−8.08; 1.3)	0.155	−1.83 (−6.31; 2.64)	0.417	−2.23 (−7.35; 2.89)	0.389	−3.67 (−8.22; 0.89)	0.113
MIX	−3.67 (−8.36; 1.03)	0.124	−0.39 (−4.86; 4.08)	0.863	−2.68 (−7.8; 2.44)	0.301	−2.18 (−6.74; 2.37)	0.343
TFV	−4.07 (−8.76; 0.62)	0.088	−2.19 (−6.66; 2.28)	0.333	−2.4 (−7.52; 2.71)	0.353	−2.85 (−7.4; 1.71)	0.218
Non-progressive								
DTG	−0.23 (−4.23; 3.76)	0.907	0.15 (−3.78; 4.09)	0.938	−0.02 (−4.09; 4.05)	0.993	−0.86 (−4.9; 3.19)	0.675
MIX	−0.74 (−4.74; 3.25)	0.713	−1.03 (−4.96; 2.91)	0.606	−0.05 (−4.12; 4.02)	0.981	−1 (−5.05; 3.04)	0.623
TFV	0.93 (−3.06; 4.93)	0.644	−0.48 (−4.41; 3.45)	0.809	0.29 (−3.78; 4.36)	0.887	−1.2 (−5.25; 2.84)	0.556
Immotile								
DTG	3.67 (−2.25; 9.6)	0.221	0.05 (−5.77; 5.88)	0.986	2.48 (−3.46; 8.41)	0.409	3.47 (−2.69; 9.62)	0.266
MIX	3.7 (−2.22; 9.62)	0.218	0.92 (−4.91; 6.74)	0.755	3.77 (−2.16; 9.71)	0.210	1.49 (−4.67; 7.65)	0.631
TFV	3.56 (−2.37; 9.48)	0.236	1.09 (−4.73; 6.92)	0.710	3.89 (−2.04; 9.83)	0.196	2.08 (−4.08; 8.23)	0.505
Circular tracks (%)								
DTG	−3.71 (−9.82; 2.4)	0.231	−2.12 (−7.8; 3.56)	0.460	−2.58 (−8.56; 3.4)	0.393	−2.57 (−7.69; 2.55)	0.321
MIX	−1.73 (−7.84; 4.37)	0.574	−1.07 (−6.75; 4.61)	0.709	−3.48 (−9.46; 2.5)	0.251	−0.89 (−6.01; 4.23)	0.731
TFV	−1.86 (−7.97; 4.25)	0.547	−3.04 (−8.72; 2.64)	0.290	−3.17 (−9.15; 2.8)	0.294	−0.98 (−6.1; 4.14)	0.705
Avg. value of speed (Motile)								
Curve speed—VCL (*µ*m/s)								
DTG	−2.3 (−9.97; 5.37)	0.553	−2.83 (−10.03; 4.37)	0.437	−1.7 (−8.56; 5.15)	0.623	−1.3 (−8.43; 5.83)	0.718
MIX	−1.11 (−8.78; 6.56)	0.774	−3.32 (−10.51; 3.88)	0.362	−4.33 (−11.18; 2.52)	0.213	−0.87 (−8; 6.26)	0.809
TFV	−1.47 (−9.14; 6.2)	0.705	−2.72 (−9.92; 4.48)	0.454	−2.23 (−9.09; 4.62)	0.519	−0.16 (−7.29; 6.97)	0.963
Avg. value—VAP (*µ*m/s)								
DTG	−0.87 (−4.51; 2.77)	0.638	−0.63 (−4.12; 2.87)	0.723	−0.32 (−3.83; 3.19)	0.856	−0.17 (−3.88; 3.54)	0.928
MIX	−0.51 (−4.15; 3.13)	0.783	−0.93 (−4.43; 2.57)	0.599	−1.54 (−5.05; 1.97)	0.385	0.04 (−3.67; 3.75)	0.983
TFV	−0.73 (−4.37; 2.91)	0.692	−0.54 (−4.03; 2.96)	0.761	−0.87 (−4.38; 2.64)	0.625	0.33 (−3.38; 4.03)	0.862
Linear speed—VSL (*µ*m/s)								
DTG	−0.47 (−3.58; 2.64)	0.764	−0.01 (−3.1; 3.09)	0.996	−0.03 (−3.24; 3.18)	0.986	0.22 (−3.16; 3.59)	0.898
MIX	−0.26 (−3.37; 2.85)	0.869	−0.45 (−3.54; 2.65)	0.774	−1 (−4.21; 2.21)	0.537	0.28 (−3.10; 3.66)	0.869
TFV	−0.53 (−3.64; 2.58)	0.735	0.12 (−2.97; 3.22)	0.937	−0.71 (−3.92; 2.5)	0.662	0.62 (−2.75; 4.00)	0.715
Straightness index—STR (%)								
DTG	−0.95 (−4.12; 2.22)	0.552	0.7 (−2.07; 3.48)	0.616	−0.71 (−4.09; 2.67)	0.676	−0.62 (−3.64; 2.39)	0.681
MIX	−1 (−4.17; 2.17)	0.532	0.54 (−2.23; 3.32)	0.698	−1.2 (−4.57; 2.18)	0.483	−0.8 (−3.82; 2.21)	0.598
TFV	−2.29 (−5.46; 0.88)	0.155	0.88 (−1.9; 3.66)	0.530	−1.5 (−4.88; 1.88)	0.380	−0.42 (−3.43; 2.59)	0.783
Linearity index—LIN (%)								
DTG	−1.01 (−4.38; 2.37)	0.555	0.74 (−2.18; 3.66)	0.616	−0.51 (−4.02; 3)	0.774	−1.23 (−4.56; 2.1)	0.465
MIX	−1.6 (−4.97; 1.78)	0.349	0.89 (−−2.04; 3.81)	0.548	−0.77 (−4.28; 2.73)	0.662	−1.15 (−4.48; 2.18)	0.494
TFV	−2.47 (−5.85; 0.9)	0.149	1.07 (−1.85; 4)	0.467	−1.42 (−4.93; 2.09)	0.422	−0.95 (−4.27; 2.38)	0.574
Oscillation index—WOB (%)								
DTG	−0.96 (−3.46; 1.53)	0.444	0.44 (−1.81; 2.7)	0.697	−0.08 (−2.64; 2.48)	0.949	−0.99 (−3.5; 1.51)	0.433
MIX	−1.57 (−4.07; 0.92)	0.213	0.88 (−1.37; 3.13)	0.440	−0.16 (−2.72; 2.4)	0.904	−0.88 (v3.38; 1.63)	0.488
TFV	−1.85 (−4.35; 0.64)	0.144	0.73 (−1.52; 2.99)	0.519	−0.86 (−3.42; 1.7)	0.508	−0.97 (−3.47; 1.53)	0.444
Avg. value of speed (Non prog.)								
Curve speed—VCL (*µ*m/s)								
DTG	0.11 (−0.7; 0.92)	0.787	0.16 (−0.61; 0.93)	0.676	−0.1 (−0.91; 0.71)	0.808	−0.06 (−0.85; 0.73)	0.886
MIX	−0.06 (−0.87; 0.76)	0.892	0.25 (−0.51; 1.02)	0.513	−0.1 (−0.91; 0.7)	0.798	0.34 (−0.45; 1.13)	0.393
TFV	−0.18 (−0.99; 0.63)	0.660	0.12 (−0.65; 0.89)	0.760	−0.27 (−1.08; 0.54)	0.513	0.31 (−0.48; 1.1)	0.437
Avg. value—VAP (*µ*m/s)								
DTG	−0.28 (−0.91; 0.35)	0.375	−0.09 (−0.61; 0.43)	0.731	−0.24 (−0.75; 0.26)	0.339	−0.27 (−0.8; 0.26)	0.311
MIX	−0.41 (−1.05; 0.22)	0.196	0.2 (−0.32; 0.72)	0.456	−0.24 (−0.75; 0.26)	0.345	−0.1 (−0.63; 0.43)	0.699
TFV	−0.48 (−1.11; 0.15)	0.135	0.06 (−0.46; 0.58)	0.818	−0.32 (−0.83; 0.18)	0.206	−0.13 (−0.66; 0.4)	0.635
Linear speed—VSL (*µ*m/s)								
DTG	−0.41 (−0.92; 0.11)	0.120	−0.03 (−0.39; 0.33)	0.868	−**0.33 (**−**0.65; 0)**	**0.049**	−**0.4 (**−**0.79; **−**0.02)**	**0.042**
MIX	−0.47 (−0.98; 0.05)	0.075	0.13 (−0.24; 0.49)	0.493	−**0.34 (**−**0.66; **−**0.01)**	**0.041**	−0.35 (−0.74; 0.04)	0.077
TFV	−**0.58 (**−−**1.09; **−**0.06)**	**0.028**	0.04 (−0.32; 0.41)	0.816	−**0.32 (**−**0.65; 0)**	**0.051**	−0.34 (−0.72; 0.05)	0.089
Straightness index—STR (%)								
DTG	−**4.91 (**−**9.08; **−**0.75)**	**0.021**	−0.55 (−3.71; 2.62)	0.732	−**3.35 (**−**6.25; **−**0.44)**	**0.024**	−**3.64 (**−**7.04; **−**0.25)**	**0.036**
MIX	−4.04 (−8.21; 0.12)	0.057	0.38 (−2.78; 3.55)	0.810	−**3.68 (**−**6.58; **−**0.78)**	**0.014**	−**5.24 (**−**8.64; **−**1.85)**	**0.003**
TFV	−**6.05 (**−**10.21; **−**1.89)**	**0.005**	−0.04 (−3.2; 3.13)	0.981	−2.33 (−5.24; 0.57)	0.114	−**4.61 (**−**8.01; **−**1.22)**	**0.008**
Linearity index—LIN (%)								
DTG	−**4.68 (**−**9.19; **−**0.17)**	**0.042**	−1.11 (−4.05; 1.83)	0.456	−**3.17 (**−**6.01; **−**0.32)**	**0.029**	−**4.6 (**−**8.37; **−**0.83)**	**0.017**
MIX	−**5.24 (**−**9.75; **−**0.73)**	**0.023**	0.53 (−2.41; 3.47)	0.723	−**3.36 (**−**6.2; **−**0.52)**	**0.021**	−**5.02 (**−**8.78; **−**1.25)**	**0.010**
TFV	−**6.02 (**−**10.53; **−**1.51)**	**0.009**	−0.15 (−3.09; 2.79)	0.920	−**3 (**−**5.84; **−**0.16)**	**0.039**	−**5.03 (**−**8.8; **−**1.27)**	**0.009**
Oscillation index—WOB (%)								
DTG	−3.75 (−7.66; 0.17)	0.061	−1.69 (−4.69; 1.31)	0.267	−2.17 (−5.03; 0.68)	0.133	−3.29 (−6.68; 0.09)	0.056
MIX	−**4.62 (**−**8.54; **−**0.7)**	**0.021**	0.51 (−2.5; 3.51)	0.739	−2.31 (−5.16; 0.54)	0.111	−**3.48 (**−**6.86; **−**0.1)**	**0.044**
TFV	−**4.49 (**−**8.41; **−**0.57)**	**0.025**	−0.3 (−3.3; 2.7)	0.844	−2.52 (−5.37; 0.33)	0.083	−**3.87 (**−**7.26; **−**0.49)**	**0.025**
Avg. value of speed (Med. prog.)								
Curve speed—VCL (*µ*m/s)								
DTG	0.37 (−9.58; 10.32)	0.941	−4.14 (−12.9; 4.62)	0.350	−1.77 (−10.69; 7.14)	0.693	0.09 (−8.47; 8.64)	0.984
MIX	1.58 (−8.37; 11.53)	0.753	−7.34 (−16.1; 1.42)	0.099	−5.2 (−14.11; 3.72)	0.250	−0.72 (−9.27; 7.83)	0.868
TFV	3.97 (−5.99; 13.92)	0.431	−4.72 (−13.47; 4.04)	0.288	−0.46 (−9.38; 8.46)	0.919	0.7 (−7.85; 9.26)	0.870
Avg. value—VAP (*µ*m/s)								
DTG	0.32 (−3.15; 3.79)	0.855	−0.96 (−3.88; 1.97)	0.517	0.01 (−2.99; 3.01)	0.996	0.68 (−2.03; 3.38)	0.621
MIX	0.37 (−3.1; 3.83)	0.834	−1.87 (−4.79; 1.06)	0.208	−0.96 (−3.96; 2.04)	0.528	0.53 (−2.17; 3.24)	0.696
TFV	1.23 (−2.23; 4.7)	0.482	−1.25 (−4.17; 1.67)	0.397	0.54 (−2.46; 3.54)	0.720	0.64 (−2.06; 3.35)	0.637
Linear speed—VSL (*µ*m/s)								
DTG	0.23 (−1.92; 2.39)	0.830	−0.3 (−2.22; 1.62)	0.754	0.19 (−1.8; 2.17)	0.853	0.73 (−1.02; 2.49)	0.408
MIX	−0.03 (−2.18; 2.13)	0.980	−0.67 (−2.59; 1.25)	0.489	−0.16 (−2.15; 1.83)	0.873	0.7 (−1.06; 2.45)	0.433
TFVv	0.39 (−1.77; 2.55)	0.721	−0.4 (−2.31; 1.52)	0.683	0.42 (−1.57; 2.4)	0.678	0.75 (−1; 2.5)	0.397
Straightness index—STR (%)								
DTG	−0.51 (−3.71; 2.69)	0.752	0.56 (−1.92; 3.05)	0.653	−0.17 (−2.65; 2.3)	0.890	−0.67 (−3.27; 1.93)	0.611
MIX	−2.08 (−5.29; 1.12)	0.200	0.48 (−2; 2.96)	0.702	0.1 (−2.38; 2.57)	0.938	−0.43 (−3.03; 2.17)	0.744
TFV	−2.46 (−5.67; 0.74)	0.130	0.32 (−2.16; 2.81)	0.796	−0.43 (−2.91; 2.05)	0.731	−0.65 (−3.26; 1.95)	0.618
Linearity index—LIN (%)								
DTG	−0.99 (−4.24; 2.27)	0.548	0.71 (−1.51; 2.94)	0.526	−0.16 (−2.46; 2.14)	0.892	−1.26 (−4.14; 1.62)	0.387
MIX	−2.68 (−5.93; 0.58)	0.106	1.43 (−0.79; 3.66)	0.204	0.45 (−1.85; 2.75)	0.700	−1.28 (−4.16; 1.6)	0.380
TFV	−3.23 (−6.48; 0.03)	0.052	0.66 (−1.57; 2.88)	0.558	−0.43 (−2.73; 1.87)	0.709	−1.34 (−4.22; 1.54)	0.358
Oscillation index—WOB (%)								
DTG	−0.71 (−3.58; 2.17)	0.626	0.99 (−1.36; 3.33)	0.405	0.45 (−1.94; 2.85)	0.708	−0.62 (−3.42; 2.19)	0.664
MIX	−2 (−4.87; 0.88)	0.171	1.86 (−0.48; 4.2)	0.118	1.11 (−1.29; 3.51)	0.361	−0.71 (−3.52; 2.09)	0.615
TFV	−2.39 (−5.27; 0.48)	0.101	0.79 (−1.55; 3.14)	0.502	0.17 (−2.23; 2.57)	0.888	−0.91 (−3.72; 1.89)	0.519
Avg. value of speed (Rapid prog.)								
Curve speed—VCL (*µ*m/s)								
DTG	−2.04 (−9.45; 5.37)	0.586	−2.49 (−10.08; 5.11)	0.517	−0.41 (−7.46; 6.64)	0.909	−2.24 (−9.13; 4.65)	0.519
MIX	−2.76 (−10.17; 4.65)	0.461	−3.26 (−10.85; 4.34)	0.396	−3.47 (−10.52; 3.58)	0.331	−2.67 (−9.56; 4.22)	0.443
TFV	−1.88 (−9.29; 5.53)	0.615	−2.01 (−9.6; 5.58)	0.600	−0.95 (−8; 6.1)	0.790	−2.11 (−9.00; 4.78)	0.544
Avg. value—VAP (*µ*m/s)								
DTG	−0.94 (−4.17; 2.28)	0.563	−0.77 (−4.09; 2.55)	0.647	0.09 (−3.27; 3.46)	0.956	−0.97 (−4.18; 2.23)	0.548
MIX	−1.45 (−4.68; 1.78)	0.374	−1.52 (−4.84; 1.81)	0.367	−1.53 (−4.9; 1.84)	0.369	−1.13 (−4.33; 2.08)	0.487
TFV	−0.77 (−3.99; 2.46)	0.638	−0.52 (−3.84; 2.8)	0.756	−0.47 (−3.84; 2.9)	0.784	−0.81 (−4.02; 2.39)	0.615
Linear speed—VSL (*µ*m/s)								
DTG	−0.89 (−3.92; 2.13)	0.560	−0.66 (−3.74; 2.43)	0.674	0.04 (−3.22; 3.29)	0.982	−0.81 (−3.94; 2.31)	0.607
MIX	−1.35 (−4.38; 1.68)	0.378	−1.46 (−4.55; 1.62)	0.348	−1.42 (−4.68; 1.83)	0.388	−0.99 (−4.12; 2.14)	0.531
TFV	−0.64 (−3.66; 2.39)	0.677	−0.44 (−3.52; 2.64)	0.777	−0.54 (−3.8; 2.72)	0.743	−0.70 (−3.82; 2.43)	0.659
Straightness index—STR (%)								
DTG	−0.05 (−0.87; 0.77)	0.909	0.06 (−0.77; 0.88)	0.895	−0.11 (−1.14; 0.92)	0.829	0.16 (−0.85; 1.16)	0.755
MIX	−0.06 (−0.88; 0.76)	0.889	−0.22 (−1.04; 0.61)	0.606	−0.1 (−1.13; 0.93)	0.842	0.11 (−0.90; 1.11)	0.833
TFV	0.1 (−0.72; 0.92)	0.812	0.07 (−0.76; 0.89)	0.872	−0.27 (−1.3; 0.76)	0.600	0.11 (−0.90; 1.11)	0.832
Linearity index—LIN (%)								
DTG	0.28 (−2.07; 2.64)	0.812	0.61 (−1.69; 2.91)	0.599	0.22 (−2.52; 2.96)	0.874	0.49 (−1.97; 2.95)	0.694
MIX	0.13 (−2.23; 2.48)	0.914	0.09 (−2.21; 2.39)	0.936	0.2 (−2.54; 2.94)	0.885	0.55 (−1.91; 3.01)	0.656
TFV	0.2 (−2.16; 2.56)	0.867	0.78 (−1.52; 3.08)	0.503	−0.07 (−2.81; 2.67)	0.961	0.43 (−2.03; 2.89)	0.730
Oscillation index—WOB (%)								
DTG	0.35 (−1.82; 2.52)	0.748	0.66 (−1.45; 2.76)	0.537	0.33 (−2.08; 2.75)	0.784	0.46 (−1.70; 2.62)	0.671
MIX	0.18 (−1.99; 2.35)	0.871	0.27 (−1.83; 2.37)	0.800	0.34 (−2.07; 2.76)	0.778	0.56 (−1.60; 2.72)	0.611
TFV	0.17 (−2; 2.34)	0.875	0.82 (−1.28; 2.92)	0.439	0.11 (−2.3; 2.53)	0.926	0.43 (−1.73; 2.59)	0.696
Avg. value of other parameters (Motile)								
Amvp. lateral head—ALH (µm/s)								
DTG	−0.06 (−0.28; 0.16)	0.568	−0.08 (−0.29; 0.12)	0.409	−0.05 (−0.25; 0.14)	0.592	−0.04 (−0.23; 0.15)	0.661
MIX	−0.04 (−0.26; 0.18)	0.745	−0.09 (−0.29; 0.12)	0.402	−0.12 (−0.31; 0.08)	0.234	−0.03 (−0.21; 0.16)	0.775
TFV	−0.05 (−0.27; 0.17)	0.664	−0.08 (−0.28; 0.12)	0.433	−0.06 (−0.26; 0.13)	0.524	−0.01 (−0.2; 0.17)	0.897
Beat frequency—BCF (Hz)								
DTG	−0.13 (−1.64; 1.38)	0.865	−0.07 (−1.6; 1.46)	0.927	−0.21 (−1.86; 1.43)	0.797	0.24 (−1.45; 1.92)	0.783
MIX	0.18 (−1.33; 1.69)	0.814	−0.08 (−1.61; 1.45)	0.917	−0.4 (−2.04; 1.25)	0.635	0.24 (−1.45; 1.93)	0.778
TFV	−0.13 (−1.64; 1.39)	0.870	0 (−1.53; 1.53)	0.998	−0.5 (−2.15; 1.14)	0.544	0.45 (−1.24; 2.14)	0.599
Avg. value of other parameters (Med. prog.)								
Amp. lateral head—ALH (µm/s)								
DTG	0.01 (−0.29; 0.31)	0.929	−0.13 (−0.39; 0.13)	0.333	−0.07 (−0.34; 0.2)	0.604	0 (−0.25; 0.26)	0.992
MIX	0.05 (−0.24; 0.35)	0.717	−0.22 (−0.48; 0.04)	0.090	−0.16 (−0.43; 0.1)	0.229	−0.03 (−0.29; 0.23)	0.816
TFV	0.12 (−0.18; 0.42)	0.426	−0.14 (−0.4; 0.12)	0.280	−0.03 (−0.3; 0.24)	0.820	0.01 (−0.24; 0.27)	0.914
Beat frequency—BCF (Hz)								
DTG	0.22 (−0.42; 0.87)	0.493	0.03 (−0.51; 0.58)	0.899	0.25 (−0.29; 0.8)	0.352	0.55 (−0.08; 1.19)	0.085
MIX	0.23 (−0.42; 0.88)	0.478	0.03 (−0.52; 0.57)	0.925	0.47 (−0.07; 1.01)	0.090	0.56 (−0.07; 1.19)	0.083
TFV	0.47 (−0.18; 1.12)	0.154	0.12 (−0.42; 0.66)	0.659	0.37 (−0.17; 0.91)	0.173	**0.65 (0.02; 1.29)**	**0.043**
Avg. value of other parameters (Rapid prog.)								
Amp. lateral head—ALH (µm/s)								
DTG	−0.04 (−0.27; 0.19)	0.736	−0.05 (−0.29; 0.19)	0.664	0 (−0.23; 0.23)	0.997	−0.05 (−0.27; 0.17)	0.630
MIX	−0.07 (−0.3; 0.16)	0.561	−0.05 (−0.3; 0.19)	0.661	−0.07 (−0.29; 0.16)	0.572	−0.06 (−0.28; 0.16)	0.581
TFV	−0.06 (−0.29; 0.17)	0.594	−0.04 (−0.28; 0.2)	0.746	−0.01 (−0.24; 0.22)	0.952	−0.05 (−0.27; 0.17)	0.672
Beat frequency—BCF (Hz)								
DTG	−0.08 (−1.21; 1.05)	0.888	−0.37 (−1.65; 0.91)	0.565	−0.45 (−1.84; 0.94)	0.522	−0.07 (−1.52; 1.38)	0.924
MIX	0.13 (−1; 1.26)	0.819	−0.44 (−1.73; 0.84)	0.492	−0.46 (−1.85; 0.93)	0.512	−0.12 (−1.58; 1.33)	0.866
TFV	0.12 (−1.01; 1.26)	0.829	−0.23 (−1.51; 1.06)	0.728	−0.32 (−1.71; 1.06)	0.645	−0.04 (−1.50; 1.41)	0.954
Hyperactive % (Motile)								
DTG	−0.64 (−3.07; 1.79)	0.601	−1.5 (−3.9; 0.9)	0.218	−0.54 (−2.82; 1.73)	0.637	−1.27 (−3.27; 0.73)	0.211
MIX	−0.69 (−3.12; 1.74)	0.573	−2.05 (−4.45; 0.35)	0.093	−1.21 (−3.48; 1.07)	0.296	−1.38 (−3.38; 0.63)	0.175
TFV	−0.33 (−2.76; 2.1)	0.786	−1.38 (−3.78; 1.02)	0.256	−0.44 (−2.72; 1.83)	0.700	−1.2 (−3.21; 0.8)	0.237
Mucous penetration % (motile)								
DTG	1.04 (−3.98; 6.07)	0.681	1.96 (−3.39; 7.31)	0.469	0.70 (−4.59; 6.00)	0.792	2.37 (−3.22; 7.96)	0.402
MIX	1.89 (−3.13; 6.92)	0.456	0.83 (−4.52; 6.18)	0.758	−0.09 (−5.38; 5.21)	0.975	2.66 (−2.93; 8.24)	0.347
TFV	1.11 (−3.91; 6.14)	0.661	1.66 (−3.69; 7.02)	0.538	−0.78 (−6.08; 4.52)	0.771	2.95 (−2.64; 8.53)	0.298
Hyperactive % (total)								
DTG	−0.61 (−2.77; 1.55)	0.573	−1.25 (−3.25; 0.75)	0.217	−0.56 (−2.45; 1.34)	0.561	−1.13 (−2.78; 0.52)	0.178
MIX	−0.72 (−2.88; 1.44)	0.509	−1.76 (−3.76; 0.23)	0.083	−1.13 (−3.02; 0.76)	0.239	−1.16 (−2.81; 0.49)	0.167
TFV	−0.42 (−2.58; 1.74)	0.700	−1.22 (−3.22; 0.77)	0.227	−0.52 (−2.42; 1.37)	0.583	−1.03 (−2.69; 0.62)	0.217
Mucous penetration % (total)								
DvTG	−0.26 (−5.77; 5.26)	0.927	1.63 (−4.34; 7.6)	0.589	−0.12 (−5.87; 5.64)	0.967	1.17 (−4.80; 7.14)	0.698
MIX	0.35 (−5.16; 5.87)	0.899	0.48 (−5.49; 6.45)	0.873	−0.82 (−6.58; 4.93)	0.777	1.86 (−4.11; 7.83)	0.537
TFV	−0.24 (−5.75; 5.28)	0.932	1.28 (−4.69; 7.25)	0.670	−1.46 (−7.22; 4.29)	0.615	2.00 (−3.96; 7.97)	0.506

*DTG* dolutegravir exposure, *TFV* tenofovir exposure, *MIX* combination antiretroviral exposure, *CI* confidence interval, *VCL* curvilinear velocity, *VAP* average path velocity, *VSL* straight-line velocity, *Rapid prog* rapid progressive sperm, *Med prog* medium progressive sperm, *Non prog* non-progressive sperm, *ALH* amplitude of lateral head displacement, *BCF* beat cross frequency

On broadly analyzing the data, no significant change was noted in the proportions of the various sperm motility subpopulations (the motile grades a–c versus the immotile grade d) during the duration of the study. However, a trend of decreasing motility and increasing proportion of immotile sperm in the drug-exposed arms compared with the control, though non-significant, is worth mentioning. On analyzing the motility and kinematic parameters of the sperm subpopulations, no significant changes were noted in the grade a during the study. Only the slower spermatozoa showed changes in motility parameter values.

Straight-line velocity (VSL) of grade c spermatozoa showed a significant decrease in the Tenofovir arm at 0 min and in all the ARV arms at 60 min compared with the control arm. A similar decrease was noted in the Dolutegravir arm at 90 min of exposure. The straightness index (STR) and Linearity Index (LIN) of the same non-progressive grade c spermatozoa showed a significant decrease at 0, 60, and 90 min after exposure in most ARV arms, while the Oscillation index (WOB) of the same non-progressive sperm sub-population was significantly decreased in the Tenofovir arm, and in the combination ARV arm at 0 and 90 min. This could be an early sign of aberrant behavior and change in path in the exposed sperm.

Beat cross frequency (BCF) of the grade b sperm population showed a significant increase at 90 min after exposure in the Tenofovir arm, and a significant decrease in the proportion of grade b sperm was also recorded at 90 min in all the antiretroviral arms.

## Discussion and limitations

In recent years, there has been a documented increase in the incidence of various reproductive disorders often attributed to chronic exposure to endocrine-disrupting chemicals (EDCs). Male factor infertility is a public health concern as its prevalence is increasing and abnormal sperm quality is linked to prenatal and postnatal pathologies [80]. EDCs can impair sperm fertilizing potential by altering their motility, causing sperm hyperactivation and acrosomal exocytosis [81]. Chronic medication, including ARVs, is increasingly being studied for their EDC effects [82]. Across continents, the common trend is HIV infection becoming a major health problem of youth entering their reproductive years [83–85]. As tens of millions of HIV-positive youth on treatment, and HIV-negative youth on PrEP, take antiretrovirals through their reproductive years, investigating the relationship between ARVs and male fertility becomes imperative.

The study results show a consistent decrease in values of VSL, STR, LIN, and WOB and an increase in BCF under Tenofovir treatment, possibly pointing to premature sperm hyperactivation. This is consistent with the previous studies documenting Tenofovir-induced changes in sperm motility and kinematics. Hyperactivated spermatozoa are likely to exhaust their energy reserves by traveling circular paths and being unable to advance toward the oocyte. The results also indicate increasing impairment of sperm motility impairment with time of exposure, with non-progressive spermatozoa showing abnormal behavior at 0, 60, and 90 min after exposure and medium progressively motile spermatozoa starting to show changes at 90 min. The trend of decreasing motility and increasing proportion of immotile sperm in the drug-exposed arms compared with the control, though non-significant, could point to a build-up of reactive oxygen species. Reactive oxygen species like superoxide anion, hydrogen peroxide, and nitric oxide are byproducts of mitochondrial oxidative metabolism and have different concentration-dependent effects on sperm. At low levels, they can trigger capacitation and hyperactivation, while at higher levels, they impair sperm motility [86].

The lack of any significant motility changes by 30 min in all sperm treatments is intriguing. ARVs have been shown to generate reactive oxygen species in other parts of the body [87, 88]. Oxidative stress is now believed to be an important cause of male infertility by impairing sperm motility and other sperm functions, with antioxidants shown to reverse these changes [89, 90]. Taurine, which is present in the semen extender Vitromed V Sperm Wash®, is a potent antioxidant with a protective influence on sperm motility [73]. This could explain the preserved motility parameters seen at 30 min. Another postulate is a biphasic response in sperm motility and other parameters on exposure to Human Tubal fluid, which is another component in the semen extender Vitromed V Sperm Wash®, where a temporary rise in motility at 30 min is lost at 60 min [79]. Only the slower spermatozoa showed changes in motility parameter values, and no significant change was noted in the grade a sub-population. This differential response of sperm subpopulations is due to the high oxidative stress and poor membrane integrity in low-motility sperm, making them more susceptible to the media they are exposed to [77–79]. Any behavior change may, therefore, first be flagged in grade b and c categories.

A trend of increasing deviation of parameter values from the control with time is noted in our study. This would suggest the possibility of greater changes with a longer exposure time. However, interpreting the results after 60 min of ejaculation would be challenging as sperm motility is known to decline in healthy men, while after 120 min, sperm viability drops [91, 92]. As this study needed additional time for sperm preparation before exposure to drugs, it was deemed unnecessary to study motility 90 min post-exposure. Additionally, this pilot study's cost and logistical limitation limited the study duration to 90 min of exposure, preventing parameters such as reactive oxygen species (ROS), Mitochondrial Membrane potential, Acrosome reaction, and sperm DNA fragmentation from being assessed.

Sperm motility and kinematics have been tied to fertility potential. Greater values for ALH, VSL, VCL, VAP, STR, WOB, and LIN are associated with better reproductive success, while the reverse is true for BCF [93]. In this study, sperm exposed to contemporary first-line antiretrovirals, both individually and in combination, showed the opposite effect in the 90 min of the analysis. Changes in the above sperm motility, kinematic parameters, and the proportion of motile sperm seen in this study may also point to the possibility of sperm DNA damage [94]. Suppose mutations and epimutations are found in exposed sperm. On further investigation, a generational toxicological investigation that investigates the risk of future generations' exposure to intergenerational and transgenerational diseases is another possibility to look into [95, 96].

The findings of this study are similar to the conclusions of other laboratory-based studies that found contemporary antiretrovirals like tenofovir, emtricitabine, and dolutegravir have deleterious effects on sperm function just like older antiretrovirals [10, 29, 31–34, 97]. The reproductive toxicity of antiretrovirals may not just be from their build-up in semen but also as early as spermatogenesis, as antiretrovirals easily cross the blood–testis barrier [98]. This conflicts with the findings of in vivo studies with many participants that randomly assigned serodiscordant couples to placebo and antiretroviral arms and compared pregnancy incidence [35, 36]. The risk of bias in these studies due to funding from a foundation and a pharmaceutical company that promotes pre-exposure prophylaxis antiretrovirals is not unnoticed. The short median duration of follow-up of the couples of 17–21 months, the lack of paternity data, the use of the same nine sites in Uganda and Kenya for data collection in both studies, and the lack of control for demographic and clinical factors of the couples do not allow for a complete assessment of the multifactorial fertility implication and intergenerational safety of chronic use of these drugs.

## Conclusion

Pharmacovigilance is a healthcare emergency, as the fast-changing world of newer drugs leaves clinicians vulnerable, as they must prescribe drugs whose long-term somatic and germline adverse effects are not fully understood. Guidelines and drugs are changing faster than we can monitor for side effects. Despite being the only mainstream first-line ARV in South Africa for 5 years, Dolutegravir’s replacement integrase inhibitor, Cabotegravir, is already being launched. More research in this field is required, especially for commonly prescribed drugs. This preliminary pilot study concludes that the current first-line ARVs used by HIV patients and HIV-negative patients on pre-exposure prophylaxis (PrEP) can alter sperm motility and kinematics [99–102]. This is the first in vitro study to document sperm motility changes by Dolutegravir alone and in combination with other ARVs. Further research with a larger sample size is warranted to quantify its impact on human fertility, addressing the limitations above, before a comprehensive conclusion of the effects of ARVs on human male fertility can be drawn. Of particular importance would be to study the impact of ARVs on reactive oxygen species levels in semen and sperm DNA fragmentation.

## Data Availability

No datasets were generated or analyzed during the current study.

## Abbreviations

HIV: Human immunodeficiency virus
ARV: Antiretroviral
PrEP: Pre-exposure prophylaxis
CASA: Computer-aided sperm analysis
SCA: Sperm Class Analyzer®
DTG: Dolutegravir
EFV: Efavirenz
TFV: Tenofovir
3TC: Lamivudine
FTC: Emtricitabine
API: Active pharmacological ingredient
DNA: Deoxyribonucleic acid
ROS: Reactive oxygen species
EDC: Endocrine disrupting chemicals
VCL: Curvilinear velocity
VSL: Straight-line velocity
VAP: Average path velocity
ALH: Amplitude of lateral head displacement
BCF: Beat cross frequency
STR: Straightness index
LIN: Linearity index
WOB: Oscillation index/wobble
MIX: Combination antiretroviral arm

## References

[1] https://cfs.hivci.org/country-factsheet.html#, accessed Jun 26 2020

[2] Bekker L-G, Rebe K, Venter F, Maartens G, Moorhouse M, Conradie F, Wallis C, Black V, Harley B, Eakle R (2016) Southern African guidelines on the safe use of pre-exposure prophylaxis in persons at risk of acquiring HIV-1 infection. South Afr J HIV Med. 10.4102/sajhivmed.v17i1.455 29568613 10.4102/sajhivmed.v17i1.455 PMC5843155

[3] https://www.nicd.ac.za/wp-content/uploads/2019/11/2019-ART-Clinical-Guidelines-25-Nov.pdf, accessed Sept 24 2020

[4] https://www.who.int/publications/i/item/update-of-recommendations-on-first--and-second-line-antiretroviral-regimens, accessed Jun 26 2020

[5] Estill J, Bertisch B (2020) More evidence for dolutegravir as first-line ART for all. The lancet HIV 7(3):e154–e155 32035042 10.1016/S2352-3018(19)30414-X

[6] Phillips AN, Venter F, Havlir D, Pozniak A, Kuritzkes D, Wensing A, Lundgren JD, De Luca A, Pillay D, Mellors J, Cambiano V, Bansi-Matharu L, Nakagawa F, Kalua T, Jahn A, Apollo T, Mugurungi O, Clayden P, Gupta RK, Barnabas R, Revill P, Cohn J, Bertagnolio S, Calmy A (2019) Risks and benefits of dolutegravir-based antiretroviral drug regimens in sub-Saharan Africa: a modelling study. The Lancet HIV 6(2):e116–e127 30503325 10.1016/S2352-3018(18)30317-5 PMC6361866

[7] Savasi V, Parisi F, Oneta M, Laoreti A, Parrilla B, Duca P, Cetin I (2019) Effects of highly active antiretroviral therapy on semen parameters of a cohort of 770 HIV-1 infected men. PLoS ONE 14(2):e0212194 30789923 10.1371/journal.pone.0212194 PMC6383866

[8] Akang EN, Dosumu OO, Ogbenna AA, Akpan U-OU, Ezeukwu JC, Odofin MO, Oremosu AA, Akanmu AS (2022) The impact of dolutegravir-based combination antiretroviral therapy on the spermatozoa and fertility parameters of men living with human immunodeficiency virus’. Andrologia 54:14621 10.1111/and.14621 PMC9722517 36261884

[9] Frapsauce C, Grabar S, Leruez-Ville M, Launay O, Sogni P, Gayet V, Viard JP, De Almeida M, Jouannet P, Dulioust E (2015) Impaired sperm motility in HIV-infected men: an unexpected adverse effect of efavirenz? Hum Reprod 30(8):1797–1806 26085581 10.1093/humrep/dev141

[10] Ahmad G, Moinard N, Jouanolou V, Daudin M, Gandia P, Bujan L (2011) In vitro assessment of the adverse effects of antiretroviral drugs on the human male gamete. Toxicol In Vitro 25(2):485–491 21130153 10.1016/j.tiv.2010.11.020

[11] Vourvahis M, Tappouni HL, Patterson KB, Chen YC, Rezk NL, Fiscus SA, Kearney BP, Rooney JF, Hui J, Cohen MS, Kashuba AD (2008) The pharmacokinetics and viral activity of tenofovir in the male genital tract. J Acquir Immune Defic Syndr 47(3):329–333 18197124 10.1097/QAI.0b013e3181632cc3 PMC2726724

[12] Patterson KB, Prince HA, Kraft E, Jenkins AJ, Shaheen NJ, Rooney JF, Cohen MS, Kashuba ADM (2011) Penetration of tenofovir and emtricitabine in mucosal tissues: implications for prevention of HIV-1 transmission. Science Translational Medicine. 10.1126/scitranslmed.3003174 22158861 10.1126/scitranslmed.3003174 PMC3483088

[13] Seifert SM, Chen X, Meditz AL, Castillo-Mancilla JR, Gardner EM, Predhomme JA, Clayton C, Austin G, Palmer BE, Zheng J-H, Klein B, Kerr BJ, Guida LA, Rower C, Rower JE, Kiser JJ, Bushman LR, Mawhinney S, Anderson PL (2016) Intracellular tenofovir and emtricitabine anabolites in genital, rectal, and blood compartments from first dose to steady state. AIDS Res Hum Retroviruses 32(10–11):981–991 27526873 10.1089/aid.2016.0008 PMC5067852

[14] Dumond JB, Reddy YS, Troiani L, Rodriguez JF, Bridges AS, Fiscus SA, Yuen GJ, Cohen MS, Kashuba AD (2008) Differential extracellular and intracellular concentrations of zidovudine and lamivudine in semen and plasma of HIV-1-infected men. J Acquir Immune Defic Syndr 48(2):156–162 18360288 10.1097/QAI.0b013e31816de21e PMC2862269

[15] Greener BN, Patterson KB, Prince HMA, Sykes CS, Adams JL, Dumond JB, Shaheen NJ, Madanick RD, Dellon ES, Cohen MS, Kashuba ADM (2013) Dolutegravir pharmacokinetics in the genital tract and colorectum of HIV-negative men after single and multiple dosing. JAIDS J Acquir Immune Defic Syndr 64(1):39–44 23945251 10.1097/QAI.0b013e31829ed7a4 PMC3804901

[16] Tseng A, Seet J, Phillips EJ (2015) The evolution of three decades of antiretroviral therapy: challenges, triumphs and the promise of the future. Br J Clin Pharmacol 79(2):182–194 24730660 10.1111/bcp.12403 PMC4309625

[17] Beyrer C, Pozniak A (2017) HIV drug resistance — an emerging threat to epidemic control. N Engl J Med 377(17):1605–1607 29069566 10.1056/NEJMp1710608

[18] Chimukangara B, Kharsany ABM, Lessells RJ, Naidoo K, Rhee S-Y, Manasa J, Gräf T, Lewis L, Cawood C, Khanyile D, Diallo K, Ayalew KA, Shafer RW, Hunt G, Pillay D, Abdool SK, De Oliveira T (2019) Moderate-to-high levels of pretreatment HIV drug resistance in KwaZulu-Natal Province, South Africa. AIDS Res Hum Retroviruses 35(2):129–138 30430843 10.1089/aid.2018.0202 PMC6360398

[19] Black V, Schwartz SR (2018) Issues about periconception use of dolutegravir are reminiscent of early concerns about efavirenz. Lancet HIV 5(12):e732–e736 30527330 10.1016/S2352-3018(18)30249-2

[20] Crawford M, Van Wyk J, Aboud M, Vannappagari V, Romach B, Curtis L, Wynne B, De Ruiter A, Smith K, Payvandi N (2020) Postmarketing surveillance of pregnancy outcomes with dolutegravir use. JAIDS J Acquir Immune Defic Syndr 83(1):e2–e5 31809366 10.1097/QAI.0000000000002213 PMC6903324

[21] Lyerly AD (2019) Dolutegravir: advancing ethical research in pregnancy. Lancet (London, England) 394(10213):1972–1974 31789207 10.1016/S0140-6736(19)32638-8

[22] Bujan L, Sergerie M, Moinard N, Martinet S, Porte L, Massip P, Pasquier C, Daudin M (2006) Decreased semen volume and spermatozoa motility in HIV-1-infected patients under antiretroviral treatment. J Androl 28(3):444–452 10.2164/jandrol.106.001529 17215546

[23] Dulioust E (2002) Semen alterations in HIV-1 infected men. Hum Reprod 17(8):2112–2118 12151446 10.1093/humrep/17.8.2112

[24] Kehl S, Weigel M, Müller D, Gentili M, Hornemann A, Sütterlin M (2011) HIV-infection and modern antiretroviral therapy impair sperm quality. Arch Gynecol Obstet 284(1):229–233 21448708 10.1007/s00404-011-1898-6

[25] Lambert-Niclot S, Poirot C, Tubiana R, Houssaini A, Soulié C, Dominguez S, Schubert B, Prades M, Bonmarchand M, Calvez V, Flandre P, Peytavin G, Marcelin A-G (2011) Effect of antiretroviral drugs on the quality of semen. J Med Virol 83(8):1391–1394 21678443 10.1002/jmv.22119

[26] Van Leeuwen E, Wit FW, Repping S, Eeftinck Schattenkerk JKM, Reiss P, Van Der Veen F, Prins JM (2008) Effects of antiretroviral therapy on semen quality’. AIDS 22(5):637–642 18317005 10.1097/QAD.0b013e3282f4de10

[27] Pavili L, Daudin M, Moinard N, Walschaerts M, Cuzin L, Massip P, Pasquier C, Bujan L (2010) Decrease of mitochondrial DNA level in sperm from patients infected with human immunodeficiency virus-1 linked to nucleoside analogue reverse transcriptase inhibitors. Fertil Steril 94(6):2151–2156 20153854 10.1016/j.fertnstert.2009.12.080

[28] Savasi V, Oneta M, Laoreti A, Parisi F, Parrilla B, Duca P, Cetin I (2018) Effects of antiretroviral therapy on sperm DNA integrity of HIV-1-infected men. Am J Mens Health 12(6):1835–1842 30132391 10.1177/1557988318794282 PMC6199444

[29] Jerónimo A, Baza MB, Río I, Vera M, Hernando V, Castilla J, Rodriguez C, Del Romero J (2017) Factors associated with seminal impairment in HIV-infected men under antiretroviral therapy. Hum Reprod 32(2):265–271 28007791 10.1093/humrep/dew321

[30] Wong N, Levy M, Stephenson I (2017) Hypogonadism in the HIV-Infected Man. Curr Treat Options Infect Dis 9(1):104–116 28344518 10.1007/s40506-017-0110-3 PMC5346114

[31] Adaramoye OA, Akanni OO, Adewumi OM, Owumi SE (2015) Lopinavir/ritonavir, an antiretroviral drug, lowers sperm quality and induces testicular oxidative damage in rats. Tokai J Exp Clin Med 40(2):51–57 26150184

[32] Oyeyipo IP, Skosana BT, Everson FP, Strijdom H, Plessis SSD (2018) Highly active antiretroviral therapy alters sperm parameters and testicular antioxidant status in diet-induced obese rats. Toxicological Research 34(1):41–48 29372000 10.5487/TR.2018.34.1.041 PMC5776917

[33] Ospina L, Álvarez-Gómez A, Cadavid A, Cardona-Maya W (2011) Tenofovir, an antiviral agent with low spermiostatic activity. Actas Urológicas Españolas (English Edition) 35(2):123–124 10.1016/j.acuro.2010.10.004 21292352

[34] Adana MY, Akang EN, Peter AI, Jegede AI, Naidu ECS, Tiloke C, Chuturgoon AA, Azu OO (2018) Naringenin attenuates highly active antiretroviral therapy-induced sperm DNA fragmentations and testicular toxicity in Sprague-Dawley rats. Andrology 6(1):166–175 29179260 10.1111/andr.12439

[35] Were EO, Heffron R, Mugo NR, Celum C, Mujugira A, Bukusi EA, Baeten JM (2014) Pre-exposure prophylaxis does not affect the fertility of HIV-1-uninfected men. AIDS 28(13):1977–1982 25259704 10.1097/QAD.0000000000000313 PMC4216472

[36] Mugo NR, Hong T, Celum C, Donnell D, Bukusi EA, John-Stewart G, Wangisi J, Were E, Heffron R, Matthews LT, Morrison S, Ngure K, Baeten JM (2014) Pregnancy incidence and outcomes among women receiving preexposure prophylaxis for HIV prevention’. JAMA 312(4):362 25038355 10.1001/jama.2014.8735 PMC4362516

[37] Sheiner EK, Sheiner E, Hammel RD, Potashnik G, Carel R (2003) Effect of occupational exposures on male fertility: literature review. Ind Health 41(2):55–62 12725464 10.2486/indhealth.41.55

[38] Salas-Huetos A, Bulló M, Salas-Salvadó J (2017) Dietary patterns, foods and nutrients in male fertility parameters and fecundability: a systematic review of observational studies. Hum Reprod Update 23(4):371–389 28333357 10.1093/humupd/dmx006

[39] Rusz A, Pilatz A, Wagenlehner F, Linn T, Diemer T, Schuppe HC, Lohmeyer J, Hossain H, Weidner W (2012) Influence of urogenital infections and inflammation on semen quality and male fertility. World J Urol 30(1):23–30 21748371 10.1007/s00345-011-0726-8

[40] Redmon JB (2002) Varicocele–the most common cause of male factor infertility? Hum Reprod Update 8(1):53–58 11866240 10.1093/humupd/8.1.53

[41] Plas E (2000) Effects of aging on male fertility? Exp Gerontol 35(5):543–551 10978677 10.1016/s0531-5565(00)00120-0

[42] Kort HI (2006) Impact of body mass index values on sperm quantity and quality. J Androl 27(3):450–452 16339454 10.2164/jandrol.05124

[43] Gaur DS, Talekar MS, Pathak VP (2010) Alcohol intake and cigarette smoking: impact of two major lifestyle factors on male fertility. Indian J Pathol Microbiol 53(1):35–40 20090219 10.4103/0377-4929.59180

[44] Sharma R, Harlev A, Agarwal A, Esteves SC (2016) Cigarette smoking and semen quality: a new meta-analysis examining the effect of the 2010 world health organization laboratory methods for the examination of human semen. Eur Urol 70(4):635–645 27113031 10.1016/j.eururo.2016.04.010

[45] Andersen JM, Herning H, Aschim EL, Hjelmesæth J, Mala T, Hanevik HI, Bungum M, Haugen TB, Witczak O (2015) Body mass index is associated with impaired semen characteristics and reduced levels of anti-Müllerian hormone across a wide weight range. PLoS ONE 10(6):e0130210 26067627 10.1371/journal.pone.0130210 PMC4466334

[46] Belloc S, Cohen-Bacrie M, Amar E, Izard V, Benkhalifa M, Dalléac A, De Mouzon J (2014) High body mass index has a deleterious effect on semen parameters except morphology: results from a large cohort study. Fertil Steril 102(5):1268–1273 25225071 10.1016/j.fertnstert.2014.07.1212

[47] Shibahara H, Obara H, Ayustawati HY, Suzuki T, Ohno A, Takamizawa S, Suzuki M (2004) Prediction of pregnancy by intrauterine insemination using CASA estimates and strict criteria in patients with male factor infertility. Int J Androl 27(2):63–68 15149462 10.1111/j.0105-6263.2004.00437.x

[48] Larsen L (2000) Computer-assisted semen analysis parameters as predictors for fertility of men from the general population. Hum Reprod 15(7):1562–1567 10875866 10.1093/humrep/15.7.1562

[49] Kashuba ADM, Dyer JR, Kramer LM, Raasch RH, Eron JJ, Cohen MS (1999) Antiretroviral-drug concentrations in semen: implications for sexual transmission of human immunodeficiency virus Type 1. Antimicrob Agents Chemother 43(8):1817–1826 10428898 10.1128/aac.43.8.1817 PMC89376

[50] Eyre RC, Zheng G, Kiessling AA (2000) Multiple drug resistance mutations in human immunodeficiency virus in semen but not blood of a man on antiretroviral therapy. Urology 55(4):591 10754183 10.1016/s0090-4295(99)00592-0

[51] Mujugira A, Baeten JM, Hodges-Mameletzis I, Haberer JE (2020) Lamivudine/Tenofovir disoproxil fumarate is an appropriate PrEP regimen. Drugs 80(18):1881–1888 33040323 10.1007/s40265-020-01419-4 PMC7710557

[52] Organization, W.H.: ‘Technical update on treatment optimization: pharmacological equivalence and clinical interchangeability of lamivudine and emtricitabine: a review of current literature: June 2012’, 2012

[53] Ford N, Shubber Z, Hill A, Vitoria M, Doherty M, Mills EJ, Gray A (2013) Comparative efficacy of lamivudine and emtricitabine: a systematic review and meta-analysis of randomized trials. PLoS ONE 8(11):e79981 24244586 10.1371/journal.pone.0079981 PMC3823593

[54] Back DJ, Burger DM, Flexner CW, Gerber JG (2005) The pharmacology of antiretroviral nucleoside and nucleotide reverse transcriptase inhibitors. JAIDS J Acquir Immune Defic Syndromes 39(Supplement 1):S1–S23 10.1097/01.qai.0000168882.67942.3f 15990598

[55] Bazzoli C, Bénech H, Rey E, Retout S, Salmon D, Duval X, Tréluyer JM, Mentré F (2011) Joint population pharmacokinetic analysis of zidovudine, lamivudine, and their active intracellular metabolites in HIV patients. Antimicrob Agents Chemother 55(7):3423–3431 21576446 10.1128/AAC.01487-10 PMC3122424

[56] Imaz A, Martinez-Picado J, Niubó J, Kashuba ADM, Ferrer E, Ouchi D, Sykes C, Rozas N, Acerete L, Curto J, Vila A, Podzamczer D (2016) HIV-1-RNA decay and dolutegravir concentrations in semen of patients starting a first antiretroviral regimen. J Infect Dis 214(10):1512–1519 27578849 10.1093/infdis/jiw406 PMC5091371

[57] Chan DJ, Ray JE (2007) Quantification of antiretroviral drugs for HIV-1 in the male genital tract: current data, limitations and implications for laboratory analysis. J Pharm Pharmacol 59(11):1451–1462 17976255 10.1211/jpp.59.11.0001

[58] Valade E, Tréluyer J-M, Illamola SM, Bouazza N, Foissac F, De Sousa Mendes M, Lui G, Chenevier-Gobeaux C, Suzan-Monti M, Rouzioux C, Assoumou L, Viard J-P, Hirt D, Urien S, Ghosn J (2015) Emtricitabine seminal plasma and blood plasma population pharmacokinetics in HIV-infected men in the EVARIST ANRS-EP 49 Study. Antimicrob Agents Chemother 59(11):6800–6806 26282407 10.1128/AAC.01517-15 PMC4604421

[59] Hendrix CW, Andrade A, Bumpus NN, Kashuba AD, Marzinke MA, Moore A, Anderson PL, Bushman LR, Fuchs EJ, Wiggins I, Radebaugh C, Prince HA, Bakshi RP, Wang R, Richardson P, Shieh E, McKinstry L, Li X, Donnell D, Elharrar V, Mayer KH, Patterson KB (2016) Dose frequency ranging pharmacokinetic study of tenofovir-emtricitabine after directly observed dosing in healthy volunteers to establish adherence benchmarks (HPTN 066). AIDS Res Hum Retroviruses 32(1):32–43 26414912 10.1089/aid.2015.0182 PMC4692123

[60] Bornman MS, Aneck-Hahn N, Bornman MS, Aneck-Hahn N (2012) The interpretation of a semen analysis. CME: Your SA Journal of CPD 30(5):163–165

[61] https://apps.who.int/iris/bitstream/handle/10665/44261/9789241547789_eng.pdf

[62] Ludwig, G., Frick, J., and Rovan, E.: ‘Spermatology : atlas and manual’ (Springer-Verlag, 1990. 1990)

[63] Aneck-Hahn NH, Schulenburg GW, Bornman MS, Farias P, De Jager C (2006) Impaired semen quality associated with environmental DDT exposure in young men living in a malaria area in the Limpopo Province, South Africa. J Androl 28(3):423–434 17192596 10.2164/jandrol.106.001701

[64] https://repository.up.ac.za/bitstream/handle/2263/53031/Moungala_Evaluating_2015.pdf?sequence=1

[65] Dorado J, Alcaraz L, Gálvez MJ, Acha D, Ortiz I, Urbano M, Hidalgo M (2013) Single-layer centrifugation through PureSperm® 80 selects improved quality spermatozoa from frozen-thawed dog semen. Anim Reprod Sci 140(3–4):232–240 23896392 10.1016/j.anireprosci.2013.06.012

[66] Morrell JM, Dalin AM, Rodriguez-Martinez H (2009) Comparison of density gradient and single layer centrifugation of stallion spermatozoa: Yield, motility and survival. Equine Vet J 41(1):53–58 19301582 10.2746/042516408x322139

[67] Glenn DR, McVicar CM, McClure N, Lewis SE (2007) Sildenafil citrate improves sperm motility but causes a premature acrosome reaction in vitro. Fertil Steril 87(5):1064–1070 17335822 10.1016/j.fertnstert.2006.11.017

[68] Whan LB, West MCL, McClure N, Lewis SEM (2006) Effects of delta-9-tetrahydrocannabinol, the primary psychoactive cannabinoid in marijuana, on human sperm function in vitro. Fertil Steril 85(3):653–660 16500334 10.1016/j.fertnstert.2005.08.027

[69] Richardson GF, McNiven MA, Mansour N (2011) Effect of methanol concentration and thaw rate on the viability and fertility of cryopreserved Arctic char, Salvelinus alpinus (L.), spermatozoa. Aquac Res 42(8):1096–1100

[70] Nynca J, Dietrich GJ, Dobosz S, Grudniewska J, Ciereszko A (2014) Effect of cryopreservation on sperm motility parameters and fertilizing ability of brown trout semen. Aquaculture 433:62–65

[71] Judycka S, Słowińska M, Nynca J, Liszewska E, Dobosz S, Ciereszko A (2020) Effects of glucose, methanol concentration, and time of equilibration on post-thaw sperm motility of rainbow trout semen. Aquaculture 520:734996

[72] Jozwiakowski MJ, Nguyen N-AT, Sisco JM, Spancake CW (1996) Solubility behavior of lamivudine crystal forms in recrystallization solvents. J Pharm Sci 85(2):193–199 8683448 10.1021/js9501728

[73] Ommati MM, Sabouri S, Retana-Marquez S, Nategh Ahmadi H, Arjmand A, Alidaee S, Mazloomi S, Akhlagh A, Abdoli N, Niknahad H, Jamshidzadeh A, Ma Y, Azarpira N, Asefi Y, Heidari R (2022) Taurine improves sperm mitochondrial indices, blunts oxidative stress parameters, and enhances steroidogenesis and kinematics of sperm in lead-exposed mice. Reprod Sci. 10.1007/s43032-022-01140-5 36484981 10.1007/s43032-022-01140-5

[74] Soler C, Yeung CH, Cooper TG (1994) Development of sperm motility patterns in the murine epididymis. Int J Androl 17(5):271–278 7698853 10.1111/j.1365-2605.1994.tb01253.x

[75] Haugen, T.B., Witczak, O., Hicks, S.A., Björndahl, L., Andersen, J.M., and Riegler, M.: ‘Sperm motility assessed by deep convolutional neural networks into WHO categories’, 2022 10.1038/s41598-023-41871-2 PMC10484948 37679484

[76] Mortimer ST, Van der Horst G, Mortimer D (2015) The future of computer-aided sperm analysis. Asian J Androl 17(4):545 25926614 10.4103/1008-682X.154312 PMC4492043

[77] Keyer S, Van Der Horst G, Maree L (2022) New approaches to define the functional competency of human sperm subpopulations and its relationship to semen quality. Int J Fertility Sterility 16(3):140–151 10.22074/IJFS.2021.531517.1132 PMC9396000 36029048

[78] Keyser, S.: ‘Sperm function in different human sperm subpopulations’, 2022

[79] Keyser S, Van Der Horst G, Maree L (2021) Progesterone, myo-inositol, dopamine and prolactin present in follicular fluid have differential effects on sperm motility subpopulations. Life 11(11):1250 34833125 10.3390/life11111250 PMC8617736

[80] Stukenborg J-B, Mitchell RT, Söder O (2021) Endocrine disruptors and the male reproductive system. Best Pract Res Clin Endocrinol Metabolism 35(5):101567 10.1016/j.beem.2021.101567 34426080

[81] Schiffer C, Müller A, Egeberg DL, Alvarez L, Brenker C, Rehfeld A, Frederiksen H, Wäschle B, Kaupp UB, Balbach M, Wachten D, Skakkebaek NE, Almstrup K, Strünker T (2014) Direct action of endocrine disrupting chemicals on human sperm. EMBO Rep 15(7):758–765 24820036 10.15252/embr.201438869 PMC4196979

[82] Archer E, Wolfaardt GM, Van Wyk JH (2017) Review: Pharmaceutical and personal care products (PPCPs) as endocrine disrupting contaminants (EDCs) in South African surface waters i. WSA 43(4):684 10.1016/j.chemosphere.2017.01.101 28187390

[83] Bekker L-G, Johnson L, Wallace M, Hosek S (2015) Building our youth for the future’. J Int AIDS Soc. 10.7448/IAS.18.2.20027 25724512 10.7448/IAS.18.2.20027 PMC4344540

[84] Nath, A.: ‘HIV/AIDS and Indian youth - a review of the literature (1980 – 2008)’, SAHARA-J: Journal of Social Aspects of HIV/AIDS, 2009, 6, (1), pp. 2–8 10.1080/17290376.2009.9724923 19399310

[85] Wilson CM, Wright PF, Safrit JT, Rudy B (2010) Epidemiology of HIV Infection and Risk in Adolescents and Youth. JAIDS J Acquir Immune Defic Syndromes 54(Supplement 1):S5–S6 10.1097/QAI.0b013e3181e243a1 PMC2924282 20571423

[86] Pereira R, Sá R, Barros A, Sousa M (2017) Major regulatory mechanisms involved in sperm motility. Asian J Androl 19(1):5–14. 10.4103/1008-682X.167716 26680031 10.4103/1008-682X.167716 PMC5227674

[87] Akay C, Cooper M, Odeleye A, Jensen BK, White MG, Vassoler F, Gannon PJ, Mankowski J, Dorsey JL, Buch AM, Cross SA, Cook DR, Peña M-M, Andersen ES, Christofidou-Solomidou M, Lindl KA, Zink MC, Clements J, Pierce RC, Kolson DL, Jordan-Sciutto KL (2014) Antiretroviral drugs induce oxidative stress and neuronal damage in the central nervous system. J Neurovirol 20(1):39–53 24420448 10.1007/s13365-013-0227-1 PMC3928514

[88] Jiang B, Hebert VY, Li Y, Mathis JM, Alexander JS, Dugas TR (2007) HIV antiretroviral drug combination induces endothelial mitochondrial dysfunction and reactive oxygen species production, but not apoptosis. Toxicol Appl Pharmacol 224(1):60–71 17669453 10.1016/j.taap.2007.06.010

[89] Dutta S, Majzoub A, Agarwal A (2019) Oxidative stress and sperm function: A systematic review on evaluation and management. Arab J Urol 17(2):87–97 31285919 10.1080/2090598X.2019.1599624 PMC6600059

[90] Vessey W, Saifi S, Sharma A, McDonald C, Almeida P, Figueiredo M, Minhas S, Virmani A, Dhillo WS, Ramsay JW, Jayasena CN (2021) Baseline levels of seminal reactive oxygen species predict improvements in sperm function following antioxidant therapy in men with infertility. Clin Endocrinol 94(1):102–110 10.1111/cen.14328 32895999

[91] Ouitrakul S, Sukprasert M, Treetampinich C, Choktanasiri W, Vallibhakara SA, Satirapod C (2018) The effect of different timing after ejaculation on sperm motility and viability in semen analysis at room temperature. J Med Assoc Thailand. 101(1):26–32

[92] Chomsrimek N, Choktanasiri W, Wongkularb A, Pratak O. Effect of time between ejaculation and analysis on sperm motility. Thai Journal of Obstetrics and Gynaecology. 2008:109–14.

[93] Fernández-López P, Garriga J, Casas I, Yeste M, Bartumeus F (2022) Predicting fertility from sperm motility landscapes. Commun Biol. 10.1038/s42003-022-03954-0 36171267 10.1038/s42003-022-03954-0 PMC9519750

[94] Aghazarian A, Huf W, Pflüger H, Klatte T (2021) Standard semen parameters vs. sperm kinematics to predict sperm DNA damage. World J Men’s Health 39:116. 10.5534/wjmh.190095 31749338 10.5534/wjmh.190095 PMC7752507

[95] Nilsson EE, Ben Maamar M, Skinner MK (2022) Role of epigenetic transgenerational inheritance in generational toxicology. Environ Epigenet. 10.1093/eep/dvac001 35186326 10.1093/eep/dvac001 PMC8848501

[96] Benazir S, Claire M, Umberto S, Mohamed B (2018) Sperm epigenome as a marker of environmental exposure and lifestyle, at the origin of diseases inheritance. Mutation Res 778:38–44. 10.1016/j.mrrev.2018.09.001 10.1016/j.mrrev.2018.09.001 30454681

[97] Akang EN, Dosumu OO, Ogbenna AA, Akpan UO, Ezeukwu JC, Odofin MO, Oremosu AA, Akanmu AS (2022) The impact of dolutegravir-based combination antiretroviral therapy on the spermatozoa and fertility parameters of men living with human immunodeficiency virus. Andrologia 54(11):e14621 36261884 10.1111/and.14621 PMC9722517

[98] Akhigbe RE, Akhigbe TM, Oyedokun PA, Famurewa AC (2024) Molecular mechanisms underpinning the protection against antiretroviral drug-induced sperm-endocrine aberrations and testicular toxicity: a review. Reproduc Toxicol 128:108629. 10.1016/j.reprotox.2024.108629 10.1016/j.reprotox.2024.108629 38825169

[99] Pike C, Rousseau E, Bekker L-G (2023) Promises and potential pitfalls of long-acting injectable pre-exposure prophylaxis. Southern Afr J HIV Med. 10.4102/sajhivmed.v24i1.1497 10.4102/sajhivmed.v24i1.1497 PMC10713495 38089889

[100] Max B (2019) Update on HIV integrase inhibitors for the treatment of HIV-1 infection. Futur Virol 14:693–709. 10.2217/fvl-2019-0077

[101] Rukmangathen R, Brahmanapalli VD, Thammisetty DP, Pemmasani D, Gali SD, Atmakuru RB (2020) Study of adverse drug reactions to antiretroviral therapy in a tertiary care hospital. Tirupati Perspect Clin Res 11(4):158–163. 10.4103/picr.PICR_133_18 33489833 10.4103/picr.PICR_133_18 PMC7819369

[102] Trifirò G, Crisafulli S (2022) A new era of pharmacovigilance: future challenges and opportunities. Front Drug Safety Regulation. 10.3389/fdsfr.2022.866898

